# Evidence for bottom‐up effects of moth abundance on forest birds in the north‐boreal zone alone

**DOI:** 10.1111/ele.14467

**Published:** 2024-12-31

**Authors:** Mahtab Yazdanian, Tuomas Kankaanpää, Thomas Merckx, Ida‐Maria Huikkonen, Juhani Itämies, Jukka Jokimäki, Aleksi Lehikoinen, Reima Leinonen, Juha Pöyry, Pasi Sihvonen, Anna Suuronen, Panu Välimäki, Sami M. Kivelä

**Affiliations:** ^1^ Ecology and Genetics University of Oulu Oulu Finland; ^2^ WILD, Biology Department Vrije Universiteit Brussel Brussels Belgium; ^3^ Nature Solutions, Finnish Environment Institute (SYKE) Helsinki Finland; ^4^ Oulu Finland; ^5^ University of Lapland Rovaniemi Finland; ^6^ Finnish Museum of Natural History University of Helsinki Helsinki Finland; ^7^ Kainuu Centre for Economic Development, Transport and the Environment Kajaani Finland; ^8^ Albus Luontopalvelut Oy Oulu Finland

**Keywords:** biotic interactions, caterpillar, forest, functional trait, insectivores, joint dynamic species distribution model, Lepidoptera, trait‐based analysis, trophic interaction

## Abstract

Insect declines are raising alarms regarding cascading effects on ecosystems, especially as many insectivorous bird populations are also declining. Here, we leveraged long‐term monitoring datasets across Finland to investigate trophic dynamics between functional groups of moths and birds in forested habitats. We reveal a positive association between the biomass of adult‐ or egg‐overwintering moths and the biomasses of resident and long‐distance migrant birds reliant on caterpillars as breeding‐season food in the north‐boreal zone. Contrary to expectations, similar signs of moth bottom‐up effects on insectivorous birds were not observed in other Finnish regions or for moths overwintering in other life stages. In fact, some negative associations between moths and birds were even detected, possibly attributable to opposite abundance trends. While supporting the existence of bottom‐up effects in the north‐boreal zone, our study emphasizes the need for further investigation to elucidate moth‐mediated trophic dynamics in areas characterized by the insect decline.

## Graphical abstract

Leveraging extensive long‐term monitoring datasets, we investigated the functional group‐level trophic dynamics between moth prey and their bird predators in Finland. Our findings suggest a bottom‐up effect of adult‐ or egg‐overwintering moths on insectivorous resident and long‐distance migrant birds in the north‐boreal zone. However, contrary to expectations, similar bottom‐up effects were not observed in other boreal zones or for moths overwintering in other life stages.

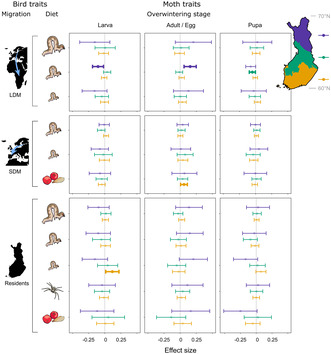

## PEER REVIEW

The peer review history for this article is available at https://www.webofscience.com/api/gateway/wos/peer‐review/10.1111/ele.14467.

## INTRODUCTION

Many insect populations are declining, mainly due to anthropogenic factors (Blumgart et al., 2022; Hallmann et al., 2017; Mancini et al., 2023; Montgomery et al., 2020; Raven & Wagner, 2021; Seibold et al., 2019; Staab et al., 2023; Ulyshen & Horn, 2023; Warren et al., 2021), yet insects are not declining everywhere (Andersson et al., 2022; Crossley et al., 2020; Pilotto et al., 2020; Yazdanian et al., 2023). Changes in insect abundance can trigger cascading effects within an ecosystem, resulting in significant consequences for ecosystem services and functions – such as pollination – either directly or through altered food‐web dynamics (Dirzo et al., 2014; Wagner, 2020; Walther, 2010). Many birds, including but not limited to specialist insectivores, depend on insect food when rearing their offspring (Tallamy & Shriver, 2021). Hence, the documented declines in insect populations have raised concerns regarding potential negative consequences for the abundance and reproductive success of insectivores (Hallmann et al., 2014; Pearce‐Higgins & Morris, 2022; Seress et al., 2018; Vesterinen et al., 2018). However, our understanding of whether and how anthropogenically induced abundance declines or natural fluctuations of insect populations cascade to higher trophic levels remains insufficiently understood. Emerging attention is drawn to this issue, but there is a trade‐off between spatial extent and accuracy of estimated trophic interactions (i.e., the feeding relationships between organisms; but see a meta‐analysis by Grames et al., 2023).

Dynamics of interacting trophic levels can be controlled bottom‐up or top‐down (Ripple et al., 2016; Terborgh, 2015). Bottom‐up control dictates that prey availability determines predator abundance, whereas under top‐down control predator abundance determines prey abundance (Ripple et al., 2016), with intraspecific density dependence potentially further impacting abundance at both trophic levels (Lack, 1954, 1966). In insect‐insectivore systems, both top‐down and bottom‐up control occur (Beilke & O'Keefe, 2023; Grames et al., 2023; Lister & Garcia, 2018). Lepidoptera and birds have been an important model system in bottom‐up trophic interaction studies focusing on single or few species at small spatial scales (Vatka et al., 2014; Visser et al., 2012) and very recently at national scale in the UK (Evans et al., 2024). Such studies have shown how insect prey abundance variation is linked to the variation in abundance of insectivorous birds (Møller, 2019; Souza‐Cole et al., 2022), as well as to their chick survival (Martay, Leech, et al., 2023) and breeding success (Grames et al., 2023; Lindström et al., 2005; Smith & Smith, 2019; Wesołowski, 2022).

Trophic interactions are fundamentally shaped by species' functional traits, determining which species interact with each other (Brousseau et al., 2018; Zakharova et al., 2019) and how susceptible these interactions are to anthropogenic drivers (Anderson et al., 2019). Functional traits are related to the performance of an organism (i.e., response trait) and/or to how an organism affects ecosystem processes (i.e., effect trait; Degen et al., 2018; Violle et al., 2007). Thus, by grouping species based on either traits that affect species' susceptibility to predation or on known trophic interactions, we can increase the signal‐to‐noise ratio of correlative analyses and formulate contrasting hypotheses for different pairs of functional groups (e.g., Spitz et al., 2014).

**FIGURE 1:**
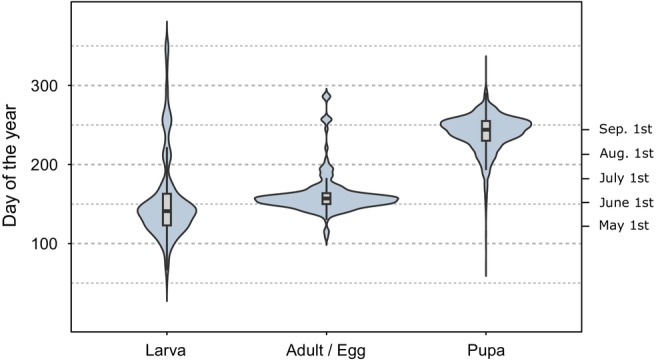
Violin plots illustrating larval phenologies of moths overwintering in different life stages. The three moth groups shown here correspond to the moth functional groups used in the analyses. The figure is based on citizen‐science data on numbers of observed larvae from www.laji.fi (Suomen Lajitietokeskus/FinBIF. http://tun.fi/HBF.52301 [downloaded 11.5.2021]).

Here, we address the functional connection between insect prey and their bird predators at a large spatial scale (i.e., Finland: ca. 340,000 km^2^) to elucidate the ecological consequences of insect abundance change at a higher trophic level. We do so by taking advantage of Finnish long‐term monitoring data on both macro‐moths and terrestrial birds. These exceptional datasets, together with advanced joint dynamic species distribution models (JDSDMs), allow us to investigate whether spatio‐temporal variation in moth abundance reflects in populations of birds that vary in their functional dependency on insect prey. We categorize bird species based on both their breeding‐season's dietary reliance on moth larvae and migration strategy affecting breeding phenology and hence the timing of peak food demand. To classify moth species, we use the overwintering stage, which affects larval temporal availability to insectivorous birds (Altermatt, 2010; Diamond et al., 2011; Figure 1). This trait‐based and both spatially and temporally extensive approach facilitates assessing the importance of insect food abundance as a driver of bird abundance change.

Specifically, we test the hypothesis that bottom‐up control drives the trophic interaction between moths and insectivorous birds, as suggested and speculated by earlier studies (Grames et al., 2023; Smith & Smith, 2019; Souza‐Cole et al., 2022; Tallamy & Shriver, 2021). This hypothesis predicts that: (i) Abundance of insectivorous birds follows moth abundance fluctuations with a time lag of 1 year, as major prey abundance effects on predator populations are realized through effects on predator breeding success, translating into population size changes after the predator's reproductive cycle (Grames et al., 2023); (ii) Moth availability to bird predators depends on moth phenology and abundance fluctuations of insectivorous birds only follow abundance fluctuations of moths available during the bird breeding season because of the importance of food availability during the nestling and fledgling phases of the bird's life cycle (Martin, 1987). Moth overwintering strategy affects their phenology so that species overwintering in the egg, larval, or adult stages are the ones that are available as caterpillars early in the bird breeding season when resident and short‐distance migrant birds are breeding (Kluen et al., 2017; Figure 1). Conversely, moths overwintering in the pupal stage appear later as caterpillars (Figure 1) and such species are expected to be available later in the bird breeding season when long‐distance migrants breed (Kluen et al., 2017); (iii) Moth abundance has a stronger influence on populations of insectivorous birds at high latitudes, where bird breeding is more time‐constrained and food webs are simpler than at low latitudes (Baker, 1939; Lack, 1950; Newth, 2004). These constraints strengthen the reliance on prey with synchronous phenology, amplifying the influence of moth abundance; (iv) Moth abundance effects are stronger on resident than on migratory birds because migratory species are also affected by prey availability and other factors along their migration routes and within overwintering areas (Ockendon et al., 2012). Our trait‐based approach using long‐term data shows that moth abundance bottom‐up effects on birds occur in the northernmost part of the boreal region.

## METHODS

### Bird abundance data

We utilized the Finnish breeding bird survey data (coordinated by the Finnish Museum of Natural History, University of Helsinki) from 1994 to 2019, gathered by using line transects and point‐count routes. Counts were executed during the peak breeding seasons specific to south‐central and northern Finland, starting around sunrise and under good weather conditions (detailed description of the survey methodology is published by Koskimies & Väisänen, 1991; Lehikoinen, 2013). We only included sites located in forested habitats to match the dominant habitat of moth monitoring sites.

In line‐transect surveys, birds were counted independently from distance along transects extending up to 6 km, with walking speeds averaging 0.75–1 km/h (Lehikoinen, 2013). We partitioned line transects into smaller habitat strips, each considered as an individual observation site. For point counts, birds were counted – independently from distance – for 5 min from predetermined fixed stations situated in habitats that were homogeneous within a 50‐m radius. Each point‐count route was composed of 20 stations, with stations being separated by at least 250 m. Each station was treated as a single observation site in the analyses. In addition to the country‐wide line‐transect and point‐count schemes, we also included point‐count data from Rovaniemi (northern Finland) from 1994 to 1995 following identical procedures, to maximize data available for analyses from the north (Jokimäki & Huhta, 1996). In all censuses, all visually and acoustically observed birds were documented. In the country‐wide monitoring schemes, the observations were converted to breeding pair counts as outlined in Koskimies and Väisänen (1991) and Lehikoinen (2013). Therefore, we multiplied the numbers of breeding pairs by two to get the number of individual birds.

All the above‐mentioned surveys were designed for monitoring birds breeding in terrestrial habitats. We exclusively focused on data collected from forest habitats and only included species known, by our expertise, to breed and/or forage in forest habitats. We deliberately excluded species for which the point count methodology does not work well, such as raptors, grouse, waders and waterfowl (Hansen et al., 2015; Lor & Malecki, 2002). Our dataset for analysing forest bird abundance variation included ~1.67 million individual birds of 78 species, from 1654 point counts and 1203 line transects (consisting of 15,768 habitat strips). Compared to number of individuals, biomass provides a better measure of a species' ecological role especially in terms of trophic interactions since biomass is directly connected to energy flow and productivity (Brown et al., 2004; Saint‐Germain et al., 2007). Therefore, we converted bird counts to biomass (grams) by multiplying the species‐specific counts with species‐specific body mass estimates obtained from del Hoyo et al. (2014). Moreover, specifying the statistical models for biomass data (see below) facilitates easier interpretation of covariate effects (Thorson, 2018).

### Bird functional groups

Based on literature, we classified forest birds according to their reliance on caterpillars in the provisioning of nestling (i.e., chick diet in precocious species), and on their migratory strategy, which also affects breeding phenology (Table 1; see list of bird species and trait references in Data S2). In caterpillar reliance (also referred to as diet), we defined five groups: (1) ‘high reliance on caterpillars’: predominantly caterpillar‐consumers, (2) ‘moderate reliance on caterpillars’: species for which a large proportion of the diet consists of caterpillars, (3) ‘low reliance on caterpillars’: species with minimal caterpillar consumption, (4) ‘other invertebrates’: species that primarily feed on other invertebrates than Lepidoptera and (5) ‘plants’: species primarily reliant on plants. Regarding migratory strategy, we categorized birds into three groups: (i) ‘residents’ (RES), (ii) ‘short‐distance migrants’ (SDM; migration within the range of the Western Palearctic) and (iii) ‘long‐distance migrants’ (LDM; migration beyond the range of the Western Palearctic). We used 11 combinations of the diet‐ and migration‐based classifications as functional groups in analyses. Note that 4 of the 15 possible combinations have no study bird. We used the pooled biomasses of bird species within each functional group as response variables.

**TABLE 1:** Bird (A) and moth (B) functional groups and their corresponding total species number and species numbers included in each zone.

	Functional group		Number of species			
	Migratory status	Caterpillar reliance/Diet	Total	North‐boreal	Mid‐boreal	South‐boreal
(A) Birds	Long‐distance migrants	High reliance	3	2	3	3
		Moderate reliance	15	14	14	13
		Low reliance	8	4	7	7
	Short‐distance migrants	Moderate reliance	15	14	13	14
		Low reliance	4	4	4	4
		Plants	4	2	4	4
	Residents	High reliance	8	7	7	8
		Moderate reliance	7	6	7	7
		Low reliance	6	3	5	5
		Other invertebrates	5	4	5	5
		Plants	3	3	3	3
	Overwintering stage					
(B) Moths		Larva	110	26	74	107
		Adult/egg	67	25	43	63
		Pupa	216	50	134	209

*Note*: For the references used in species classification, see the list of bird and moth species in Data S2 and S3.

### Moth abundance data

We used macro‐moth abundance data from the Finnish National Moth Monitoring Scheme (Nocturna), which has extensive spatial coverage (~280 locations) since 1993 (Leinonen et al., 2017) and from the Värriö Nocturnal Moth Monitoring Scheme, collected at the Värriö research station in northeastern Finland since 1978 (Hunter et al., 2014). Both monitoring schemes use light‐traps to sample the entire adult moth activity season, roughly from April to October. We exclusively considered data from 85 traps (nine of which from Värriö) located within forest‐dominated landscapes that had been sampled for a minimum of 5 years during 1993–2019, reducing habitat variability and eliminating biases from short‐running traps.

We transformed the annual trap‐specific counts of moth individuals into annual fresh biomass (mg), for the reasons explained above for birds, by multiplying the yearly counts of individuals – for each species and trap – by estimates of the species‐specific body mass deriving from a linear regression model that considered species wingspan (mm) and body plan (stout or slender) (Kohonen, 2020). The regression model was based on empirical data derived from 1542 specimens spanning 164 genera (see Yazdanian et al. (2023) for details). The strong positive correlation between annual estimates for counts and biomass of moths in our data (*r* = 0.989) suggests that the choice between biomass and counts does not affect results.

### Moth functional groups

Using ecological and life‐history traits, we categorized all moth species based on their susceptibility to bird predation. We used larval traits (compiled using Pöyry et al., 2017 and references provided in Data S3) to classify moths as either ‘easy’ or ‘difficult’ prey. The ‘easy’ category comprised diurnally active caterpillars with short, soft, no or only minimal hairs, without a hiding lifestyle. Cryptic, aposematic, hairy, nocturnal and hiding caterpillars were classified as ‘difficult’. We assumed that the ‘easy’ moths are main prey of insectivorous birds, so we only included them in the analyses (cf. Remmel et al., 2011). The ‘easy’ group Included 586,608 moth individuals, encompassing 393 species. We further divided the ‘easy’ moths into three functional groups based on the overwintering stage – larva, adult or egg and pupa (Table 1) – because the overwintering life stage affects the temporal availability (i.e., phenology) of larvae (Figure 1), which is crucial for trophic interactions with birds.

### Climate data and bioclimatic regions

Variations in temperature and precipitation can directly and indirectly (i.e., through food availability) affect the breeding success of woodland birds (Leech & Crick, 2007; Meller et al., 2018). Accordingly, change in breeding temperature affects abundance trends of long‐distance migratory birds (Martay, Pearce‐Higgins, et al., 2023) and precipitation and drought events may be even more important drivers of avian abundance than temperature per se (Grinde et al., 2017). Hence, we used spatially interpolated temperature and precipitation data during the bird breeding season in Finland (May–June) at a 10 km grid resolution (Finnish meteorological institute; https://paituli.csc.fi/, 2021). We computed the annual anomalies of mean temperature and total precipitation for the period 1992–2018. We used the geographical centroid of each habitat strip in line transects and point coordinates for point counts to extract climatic values. We incorporated the anomaly of climate values from the preceding year as covariates, as we anticipate that breeding season conditions affect breeding success and thus population size in the subsequent year (Oedekoven et al., 2017). As Finland extends over ca. 10 latitudinal degrees, we divided the country into three subzones of the boreal (taiga) biome according to the traditional forest vegetation division (Ahti et al., 1968): north‐boreal (NB), mid‐boreal (MB) and south‐/hemi‐boreal (hereafter south‐boreal: SB) and repeated the analysis for each of them to avoid model convergence issues.

### Statistical analyses

We employed JDSDMs to analyse bird and moth biomass fluctuations and the covariation in their biomass changes over space and time. These models were fitted as vector‐autoregressive spatio‐temporal models using the VAST package (version 3.10.1; Thorson, 2019; Thorson & Barnett, 2017) in the R environment (version 4.2.1; R Core Team, 2022). JDSDMs, as implemented in VAST, allow for the simultaneous analysis of spatial and spatio‐temporal variations in population densities across multiple species, while considering both spatial and temporal autocorrelations. Additionally, they consider the influence of environmental factors on the occurrence and abundance of species (Thorson et al., 2016; Thorson & Barnett, 2017).

To assess the bottom‐up effects of moths on birds, we interpolated yearly ln‐anomalies of moth biomass for each bird observation site (details in Appendix S1). We checked the correlation between observed and interpolated biomass values (overall, *r* > 0.66; *r* ≥ 0.72 in eight out of nine region‐moth‐group combinations; Table S1) to ensure that our interpolation procedure is accurate and does not introduce biases into our modelling (see also Figure S1). In our multivariate JDSDMs for birds, we used the biomass of 11 bird functional groups as response variables. Based on the input of observed biomass of bird functional group *c* (*c* = {LDM_Low_Reliance, …, SDM_Plants}) at site *s* (*s* = 1, …, *n*_sites_; *n*_sites_ equals 3571 in NB, 4885 in MB, 8199 in SB) and year *t* (*t* = 1994, …, 2019), *b*(*s*, *c*, *t*), VAST estimates biomass density (biomass per unit area; model output), *d*(*s*, *c*, *t*). The ln‐transformed biomass anomalies of the three moth functional groups from the previous year, as well as mean temperature anomaly and total precipitation anomaly from the previous year were set as covariates in the analyses. Through sensitivity analyses (Appendix S2) we determined that a 1‐year time lag between adult moth biomass and bird biomass was ideal for our study. All the covariates were standardized prior to analyses (i.e., subtracted by mean and divided by standard deviation).

We configured the VAST model as a Poisson‐link delta model, which closely approximates a Tweedie distribution to enhance computational efficiency and to facilitate easy interpretation of covariate effects (Thorson, 2018). In delta models, the probability distribution for the biomass data *b*(*s*, *c*, *t*) is broken down into occurrence probability of functional group *c*, at location *s* in year *t* (i.e., at least one species belonging to group *c* is present at site *s* in year *t*), *r*₁(*s*, *c*, *t*) and the biomass of functional group *c*, at location *s*, in year *t* conditional on occurrence, *r*₂(*s*, *c*, *t*). We specified Gamma distribution for bird biomass, *b*, as.

(1) $$\mathrm{Pr}\left(b_{s,c,t}=B\right)=\left\{\begin{array}{cc} 1-r_{1}\left(s,c,t\right) & \text{if}\;B=0\\ r_{1}\left(s,c,t\right)\times \text{gamma}\;\left\{B|r_{2}\left(s,c,t\right),\sigma_{m}^{2}\;\left(c\right)\right\} & \;\text{if}\;B>0 \end{array}\right.$$

where $\sigma_{m}^{2}$ is the residual variance in biomass. In the Poisson‐link configuration, we can express *r*₁(*s*, *c*, *t*) as a function of density, *N*(*s*, *c*, *t*) (i.e., the number of individuals per unit area) and *r*₂(*s*, *c*, *t*) as a function of both *N*(*s*, *c*, *t*) and average species body mass, *W*(*s*, *c*, *t*) (i.e., the mean body mass of a species in group *c*) which are modelled through two linear predictors, both of which include covariate effects, terms for spatial and spatio‐temporal variation, overdispersion (i.e., census route identity) and bird census type (Appendix S3). Moreover, the linear predictor for *N*(*s*, *c*, *t*) included a first‐order temporal autocorrelation (AR1) for functional‐group‐specific intercepts for each year. We set functional‐group‐specific intercepts, variances and latent factor loadings in spatial, spatio‐temporal and overdispersion variation, effects of environmental and bird census type covariates and the parameters governing spatial autocorrelation (see Appendix S3) as fixed effects. Spatial, spatio‐temporal and overdispersion factors were treated as random effects.

Fixed effects were estimated by maximizing the log‐marginal likelihood (Laplace approximation; Skaug & Fournier, 2006) through the Template Model Builder R package (Kristensen et al., 2016), while integrating over random effects. Standard errors were calculated using a generalized form of the delta method. Random effects were predicted based on the joint likelihood of random effects and data, with respect to the maximum likelihood estimates of the fixed effects (see Thorson et al., 2015 for details). We assessed the goodness‐of‐fit of the models by examining diagnostic plots generated by using tools in the R package “DHARMa” (Hartig & Hartig, 2020) and calculated the proportion of deviance explained by using tools in the VAST package to measure model explanatory power (Table S2). We corrected the risk level for Type I errors (false positives) arising from conducting multiple statistical tests (see Appendix S4 for details). Thus, we considered parameters with 99.8% instead of 95% confidence intervals excluding zero to be statistically significant. We found some effects that were marginally non‐significant (but see Amrhein et al., 2019), but stronger than an effect that was significant at 99.8% confidence level in same combination of region moth functional group. To assess the probability of finding these high effect sizes by chance, we calculated the probability of finding the observed number of effects with sizes equal to or greater than the lowest effect that was statistically significant in the strict sense within each moth‐group‐region combination. We used an empirical cumulative distribution function based on the distribution of effect sizes produced by our analyses when deriving the probabilities for the high effects (details in Appendix S4). We only present and interpret the results from the first (density) linear predictor (Table S3) because only they are meaningful for assessing the study hypothesis (see Table S4 and Figures S2 and S3 for the average body mass linear predictor).

## RESULTS

**FIGURE 2:**
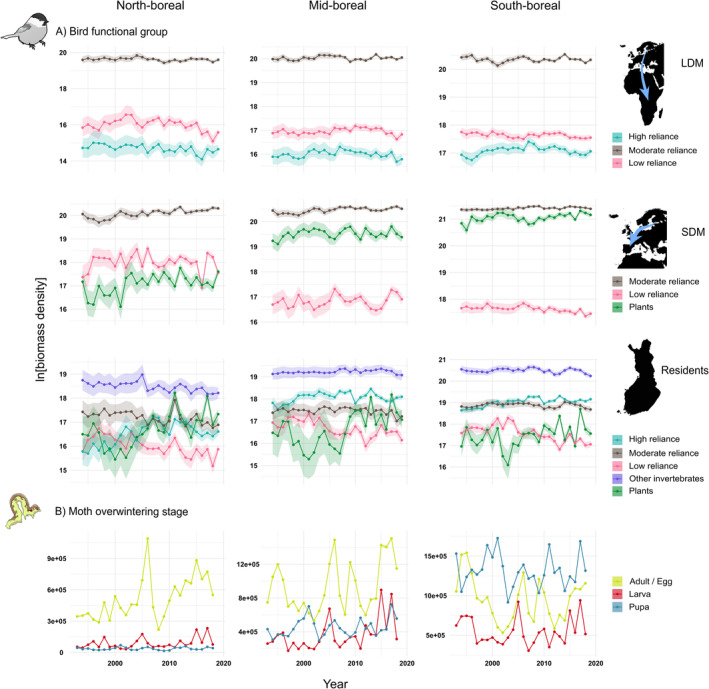
Temporal trends in ln‐transformed biomass density of bird functional groups (rows 1–3) and in absolute biomass density of moth functional groups (bottom row) in the north‐boreal (left column), mid‐boreal (middle column) and south‐boreal (right column) regions of Finland. Error strips (colour shading) indicate 95% confidence intervals.

**FIGURE 3:**
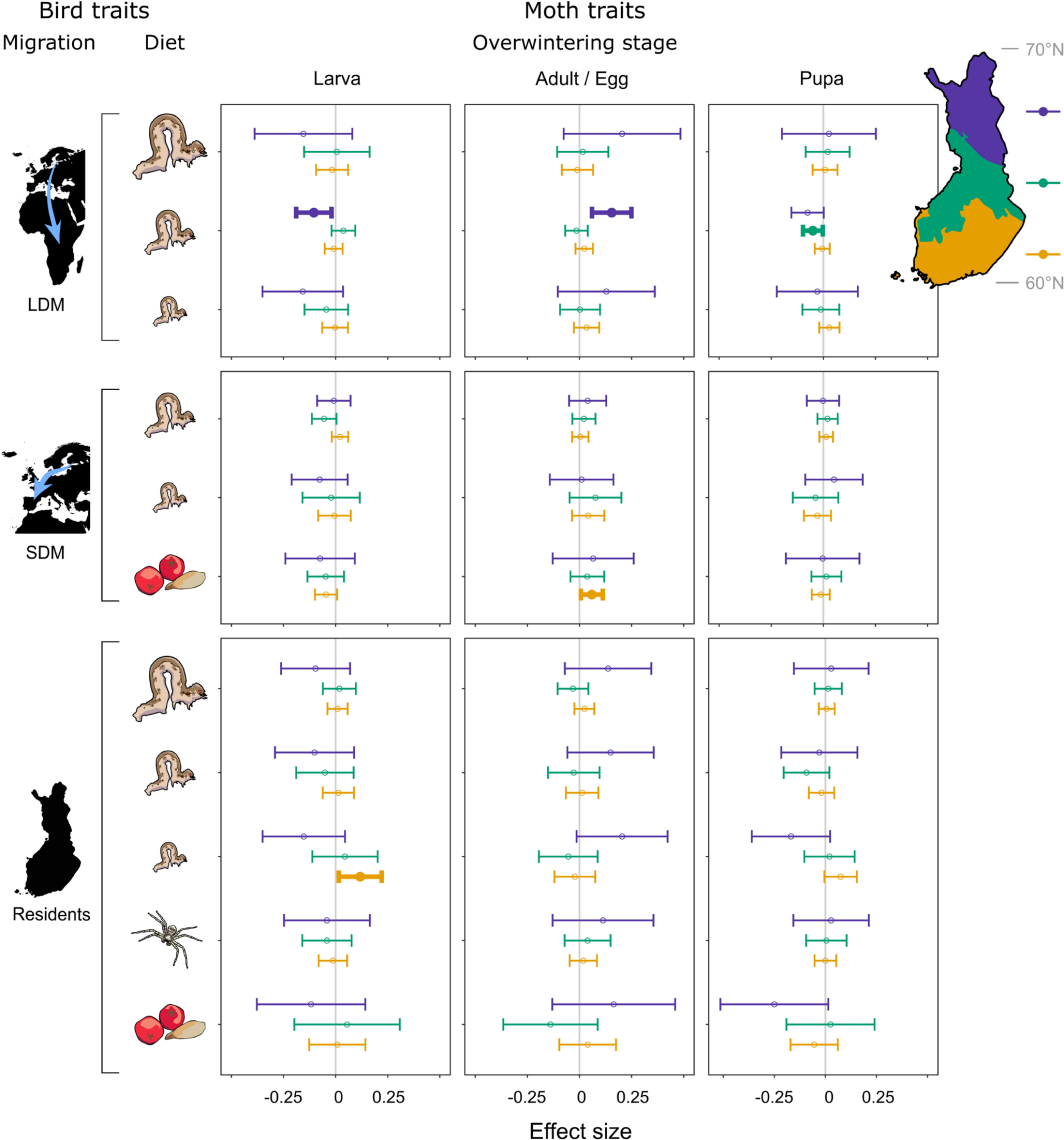
Estimated association of the ln‐transformed moth biomass anomaly of the previous year of larval‐overwintering moths (left column), adult‐/egg‐overwintering moths (middle column) and pupal‐overwintering moths (right column) with the biomass density of bird functional groups (rows) in north‐boreal (violet), mid‐boreal (green) and south‐boreal (orange) regions of Finland. Bird migratory status consists of Residents, short‐distance migrants (SDM), and long‐distance migrants (LDM). For bird diet, the reliance of birds on caterpillars is indicated by caterpillar size (i.e., a larger caterpillar links to higher reliance), the spider represents reliance on other invertebrates and the rowanberries and the pine seed represent reliance on plants as food. Points stand for the estimated effect size, while whiskers are confidence intervals adjusted for multiple testing calculated as 100 × (1 − [0.05/2 × 11])% = 99.8%. Significant effects (*α* = 0.002) are shown with bold whiskers and filled points.

**FIGURE 4:**
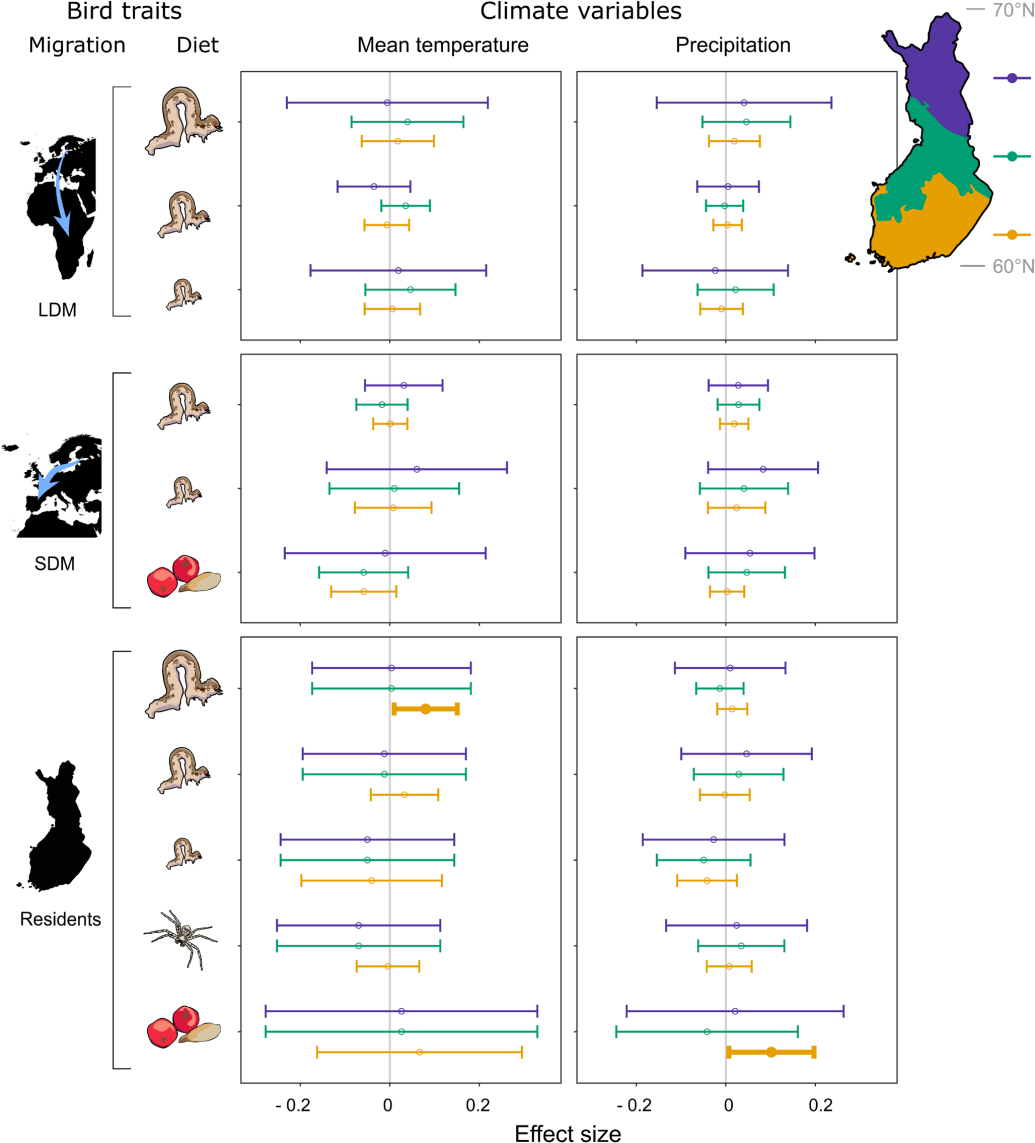
Estimated association of the previous year anomaly of mean temperature (left column) and precipitation sum (right column) during the bird breeding season with the biomass density of bird functional groups (rows) in north‐boreal (violet), mid‐boreal (green) and south‐boreal (orange) regions of Finland. See Figure 3 for information on symbols, points and whiskers.

Biomass density of both bird and moth functional groups fluctuated across space and time in Finland (Figure 2). There was some co‐variation between moth and bird biomasses, as indicated by five moth biomass effects on birds that were strongly statistically supported (Figure 3). Only two climate effects on bird biomass were supported at the 99.8% confidence level (Figure 4).

There was a positive adult‐/egg‐overwintering moth abundance effect on the abundance of long‐distance migrant birds moderately reliant on caterpillars in the north‐boreal region (Figure 3; Table S3). In this region, there were also stronger positive adult‐/egg‐overwintering moth effects on long‐distance migrants with a high reliance on caterpillars and on residents with a low reliance on caterpillars, with these effects having confidence intervals that only marginally encompassed zero (Figure 3; Table S3), which suggests real effects (cf. Amrhein et al., 2019). Indeed, the probability of getting, by chance, three effects with an absolute value equal to or greater than the one significant at the 99.8% confidence level from the empirical distribution of effect sizes produced by this study is only 0.0002.

In the south‐boreal region, there were positive effects of larval overwintering moths on resident birds with a low reliance on caterpillars and adult‐/egg‐overwintering moths on plant‐eating short‐distance migrant birds (Figure 3; Table S3) and no other effects stronger than these, resulting in probabilities of >0.1 of getting these single effects from the empirical distribution of effect sizes by chance.

There was a negative effect of larval overwintering moths on long‐distance migrant birds moderately relying on caterpillars in the north‐boreal region and similar stronger effects, but with confidence intervals marginally encompassing zero, on the two other long‐distance migrant groups and on residents with a low reliance on caterpillars (Figure 3; Table S3). The calculated probability of obtaining at least four similarly strong effects from the empirical distribution of effect sizes by chance is 0.0042. Finally, there was a negative effect of pupal overwintering moths on long‐distance migrant birds moderately reliant on caterpillars in the mid‐boreal region and a stronger but marginally zero‐encompassing effect on residents moderately reliant on caterpillars in the mid‐boreal region (Figure 3; Table S3). The probability of getting these two effects by chance is 0.3333.

In general, moth biomass had a more consistent impact on bird biomass than climate variables. Previous breeding season's mean temperature anomaly was positively associated with resident birds highly reliant on caterpillars only in the south‐boreal region (Figure 4; Table S3). Previous breeding season's precipitation anomaly had a positive association only with resident plant‐eating birds in the south‐boreal region (Figure 4; Table S3).

## DISCUSSION

Abundance of resident and long‐distance migrant birds followed moth abundance with the predicted time lag of 1 year, but only in the northernmost (i.e., north‐boreal) region and this was only the case for moths overwintering in the egg or adult stage, suggesting that early‐season moths are important for birds, especially in the north. This group of moths has both high dominance and large population fluctuations in the north and becomes relatively less abundant and stable towards the south (Figure 2). Phenologically, this group is relatively early, typically matching larval emergence with budburst of the host plant (Figure 1). As the estimated bottom‐up effects were positive on all resident and long‐distance migrant birds, the evidence of bottom‐up effects is relatively strong in the north‐boreal region. Hence, the results suggest that bottom‐up effects are relatively stronger towards these northern environments, where time constraints on bird breeding are intense (Baker, 1939; Lack, 1950) and food webs are relatively simple (Newth, 2004). Alternatively, our approach could reveal such effects only in the north due to its prominence of moth population fluctuations. Either way, the results are consistent with our prediction that moth abundance has a stronger bottom‐up effect on insectivorous birds at high than at low latitudes. Finally, the results are inconsistent with the prediction that resident birds would be subject to stronger bottom‐up effects than migratory birds, because the bottom‐up effects in the north‐boreal region pertained to both resident and migratory birds. Hence, the hypothesis that populations of migratory birds are less affected by food availability in their breeding grounds than populations of resident birds is refuted in this study.

Bottom‐up effects may be more pronounced at high than at lower latitudes because temporal variation in moth abundance is higher in the far north (Figure 2), which is the case especially for moths overwintering as eggs, including species that can show outbreaks in the subarctic region (Jepsen et al., 2008; Klemola et al., 2006; Leinonen et al., 2017). Besides the ecological consequences of high moth abundance fluctuations in the north, such fluctuations also result in a better signal‐to‐noise‐ratio for estimating the covariate effect, which is likely to result in more statistically significant effects in the north. Owing to the early larval phenology of larval‐overwintering moths, we expected similar bottom‐up effects also by this moth group, especially in the north, but such effects were not found, except one in the southernmost study region (on resident birds minimally dependent on caterpillars). This general lack of bottom‐up effects by larval‐overwintering moths may be because of less fluctuating population dynamics in this moth group in the north, or because they are temporally able to avoid bird predation, at least in less time‐constrained environments in mid‐ and south‐boreal regions, by pupating before the peak bird breeding season.

In fact, the effects of both larval and pupal overwintering moths on bird biomass were negative in the north‐boreal region. This may be, for example, due to effects of winter or spring conditions on annual phenological matching of trophic levels and moth abundance. Alternatively, the unexpected negative associations between larval‐overwintering moths and birds in the north stem from the stable abundance of larval‐overwintering moths (Yazdanian et al., 2023) and declining abundance of some bird functional groups (Figure 2).

While caterpillars of moths overwintering in the larval stage or as adult/egg typically become available before those of pupal‐overwintering moths, we would still have expected clearer bottom‐up effects by pupal‐overwintering moths on insectivorous birds. This expectation follows the observation that bird predation on caterpillars peaks in mid‐June in Estonia (Remmel et al., 2009) and caterpillars of pupal‐overwintering species are already available that time there, a bit to the south of our study area. This suggests that also caterpillars of pupal‐overwintering species would contribute to potential bottom‐up effects on insectivorous birds. Yet, caterpillars of pupal‐overwintering moths are relatively smaller than those of moths overwintering in the other life stages in mid‐June, which may reduce bird predation on them (except for species overwintering as pupal imagos, whose larvae emerge as early as egg‐overwinterers; however, pupal imago‐overwinterers comprise only 19 species in our data). There were also some negative pupal‐overwintering moth associations with resident and long‐distance migrant birds with low to moderate reliance on caterpillars and plant‐eating birds in north‐ and mid‐boreal regions, probably as a result of opposing abundance trends (compare Figure 2 with Yazdanian et al., 2023). Climate variables did not explain bird abundance variation in this study, except a positive temperature and precipitation effect in the south‐boreal region, suggesting that short‐term weather events may affect bird breeding success more than longer‐term climatic conditions (Radford & Du Plessis, 2003; Wright et al., 2009) or that the climate effect on bird breeding success is only weak at the functional group level. Nonetheless, breeding season temperature had a positive effect on productivity of common Finnish passerines in a previous study (Meller et al., 2018).

Several methodological and biological factors may have affected our ability to detect bottom‐up effects. First, potential inaccuracies in assumed time lags may have confounded results. We estimated caterpillar abundance based on adult moth abundance, but whether caterpillar abundance better correlates with the abundance of adults in the parental (i.e., previous) adult generation or the abundance of adults in the subsequent generation (i.e., the generation where the larvae belong to) remains uncertain and context‐specific. However, a sensitivity analysis of time lags in the north‐boreal region supported the choice of a one‐year time lag (Appendix S2). Second, effects may be mediated via moth host plant use; adult‐/egg‐overwinterers predominantly feed on trees and larval‐overwinterers primarily feed on understory plants (Figure S4; Virtanen & Neuvonen, 1999). Consequently, the foraging strategies of birds might affect with which moth groups they interact. Third, slight habitat differences between moth trap and bird monitoring sites and the broad categorization of both birds and moths into functional groups may have introduced inaccuracies. Also, species foraging in different macro‐ and micro‐habitats are likely grouped together in this approach. Fourth, other factors than breeding period food availability affect bird abundance (e.g., availability of nest sites, predation, or winter mortality). This is likely the case in Finland because moth biomass has remained stable or even increased (Yazdanian et al., 2023). Thus, abundance of insectivorous birds may not be limited by availability of moth prey in the breeding grounds, provided that the current moth abundance exceeds the needs of birds. Fifth, potential opposite abundance changes of bird species included in the same bird group could result in a constant group‐specific abundance and hence mask bottom‐up effects on some species.

Studies of insect abundance changes have largely focused on a single trophic level (Hallmann et al., 2017; Wagner et al., 2021; Wepprich et al., 2019), although it is evident that abundance changes in one trophic level may cascade on abundances at other trophic levels (Lister & Garcia, 2018; Møller, 2019; Souza‐Cole et al., 2022; Tallamy & Shriver, 2021). Some loose connections between abundance changes of prey and predators – suggesting bottom‐up effects – have been made by combining different monitoring data (Evans et al., 2024; Lister & Garcia, 2018; Wesołowski, 2022). Our innovative approach addressed moth prey abundance impacts on bird predator abundances at a large spatial scale, revealing bottom‐up effects in the northernmost region where moth abundance fluctuates the most. Our result is likely based on natural moth abundance fluctuations since there has not been an overall moth abundance decline in Finland during the study period (Yazdanian et al., 2023). Future research should investigate these dynamics also under scenarios of substantial insect abundance decline, contrasting regions varying in the magnitude of insect decline. This will elucidate the extent of bottom‐up effects and their implications for predator populations under varied ecological contexts. Additionally, studying long‐term dynamics of birds and their prey at the same locations would facilitate a more direct analysis of the dynamics of trophic interactions (see e.g., Holmes, 2011; Holmes & Likens, 2016). While our study delved into the bottom‐up effects of moths on birds, investigating top‐down effects of birds on insect abundance is also essential for a comprehensive understanding of predator–prey dynamics.

## AUTHOR CONTRIBUTIONS

M.Y. designed the study, led the analyses and investigation, contributed equally to conceptualization, data curation, methodology, validation and visualization and prepared the original manuscript draft. T.K. equally contributed to conceptualization, data curation, validation and visualization and provided a supporting role in the formal analysis and investigation. T.M. contributed equally to conceptualization and validation and supported the investigation. I.‐M.H., J.I., J.J., R.L., A.L., J.P., P.S., A.S. and P.V. provided resources. S.M.K. contributed equally to conceptualization, methodology and validation, provided support in data curation, formal analysis and investigation, led the funding acquisition, project administration and supervision and supported the preparation of the original draft. All authors contributed to review and editing of various draft versions and the revision of the manuscript.

## Supporting information


Data S1.

Data S2.

Data S3.

Appendix S1.

Appendix S2.

Appendix S3.

Appendix S4.

## Data Availability

The data underlying the results of this study, R scripts of analyses and the model outputs (i.e., fit objects to reload the fitted model) are openly available in Zenodo at https://doi.org/10.5281/zenodo.11505520 (Yazdanian, 2024).
