# Supplementary material for: Evidence for bottom‐up effects of moth abundance on forest birds in the north‐boreal zone alone
Source: Ecol Lett. 2024 Dec 31;27(12):e14467. doi: 10.1111/ele.14467 (PMC11686949; doi:10.1111/ele.14467)
Supplement: Supplementary file 1 — Data S1. [file ELE-27-0-s003.docx]

**Supporting Information**

**Table S1. Correlation between interpolated biomass values and observed biomass values at trap locations.**

| *Zone* | *Moth group* | *Correlation coefficient* |
| --- | --- | --- |
|  | Larva | 0.79 |
| North-boreal | Adult/Egg | 0.84 |
|  | Pupa | 0.78 |
| Mid-boreal | Larva | 0.80 |
|  | Adult/Egg | 0.88 |
|  | Pupa | 0.72 |
| South-boreal | Larva | 0.76 |
|  | Adult/Egg | 0.84 |
|  | Pupa | 0.66 |

**Table S2. Model performance assessment using tools provided in the VAST R package.** The proportion of explained deviance is calculated as one minus the deviance of the target model divided by the deviance of the null model.

| *Models* | *Deviance of target model* | *Deviance of null model* | *Proportion of explained deviance* |
| --- | --- | --- | --- |
| North-boreal model | 91806.9 | 122797.3 | 0.253 |
| Mid-boreal model | 157668.8 | 188604.7 | 0.164 |
| South-boreal model | 382432.8 | 422040.2 | 0.094 |
| Moth model | 1917.422 | 6988.651 | 0.726 |

**Table S3. Covariate effects on bird functional groups estimated for density of individuals (first linear predictor; density of individuals).** Covariates are ln[moth biomass anomaly of previous year] of larval-overwintering moths, adult- / egg-overwintering moths, pupal-overwintering moths, and previous year anomaly of mean temperature and precipitation sum during bird breeding season in north-boreal, mid-boreal, and south-boreal regions in Finland. Lower and upper 99.8% confidence limits (LCI and UCI, respectively; adjusted for multiple testing calculated as 100 × (1 – (0.05/2×11))% = 99.8%) of the estimated covariate effects are given.

|  |  |  | Moth overwintering stage | | | | | | | | | Climatic covariates | | | | | |
| --- | --- | --- | --- | --- | --- | --- | --- | --- | --- | --- | --- | --- | --- | --- | --- | --- | --- |
| Zone  (Models) | **Migratory status** | **Caterpillar reliance/Diet** | **Larva** | | | **Adult/Egg** | | | **Pupa** | | | **Temperature** | | | **Precipitation** | | |
|  |  |  | **Estimate** | **LCI** | **UCI** | **Estimate** | **LCI** | **UCI** | **Estimate** | **LCI** | **UCI** | **Estimate** | **LCI** | **UCI** | **Estimate** | **LCI** | **UCI** |
| North boreal | **Resident** | **High reliance** | -0.0962 | -0.2615 | 0.0692 | 0.1372 | -0.0702 | 0.3445 | 0.0286 | -0.1541 | 0.2113 | 0.0038 | -0.1735 | 0.1811 | 0.0097 | -0.1139 | 0.1332 |
|  |  | **Moderate reliance** | -0.1011 | -0.2908 | 0.0886 | 0.1492 | -0.0579 | 0.3563 | -0.0298 | -0.2161 | 0.1566 | -0.0123 | -0.1946 | 0.1700 | 0.0462 | -0.0996 | 0.1921 |
|  |  | **Low reliance** | -0.1526 | -0.3504 | 0.0451 | 0.2045 | -0.0138 | 0.4228 | -0.1683 | -0.3601 | 0.0234 | -0.0500 | -0.2439 | 0.1440 | -0.0274 | -0.1856 | 0.1309 |
|  |  | **Other invertebrates** | -0.0418 | -0.2477 | 0.1642 | 0.1129 | -0.1289 | 0.3548 | 0.0279 | -0.1567 | 0.2125 | -0.0696 | -0.2521 | 0.1128 | 0.0240 | -0.1334 | 0.1815 |
|  |  | **Plants** | -0.1177 | -0.3778 | 0.1423 | 0.1643 | -0.1304 | 0.4589 | -0.2498 | -0.5139 | 0.0143 | 0.0262 | -0.2773 | 0.3297 | 0.0207 | -0.2216 | 0.2631 |
|  | **Short-distance** | **Moderate reliance** | -0.0084 | -0.0886 | 0.0718 | 0.0393 | -0.0497 | 0.1283 | -0.0017 | -0.0792 | 0.0757 | 0.0312 | -0.0554 | 0.1178 | 0.0279 | -0.0383 | 0.0942 |
|  |  | **Low reliance** | -0.0766 | -0.2110 | 0.0579 | 0.0104 | -0.1423 | 0.1631 | 0.0512 | -0.0867 | 0.1891 | 0.0605 | -0.1409 | 0.2619 | 0.0832 | -0.0396 | 0.2060 |
|  |  | **Plants** | -0.0745 | -0.2412 | 0.0921 | 0.0657 | -0.1285 | 0.2600 | -0.0033 | -0.1797 | 0.1732 | -0.0102 | -0.2346 | 0.2141 | 0.0538 | -0.0905 | 0.1980 |
|  | **Long-distance** | **High reliance** | -0.1542 | -0.3884 | 0.0800 | 0.2045 | -0.0754 | 0.4844 | 0.0268 | -0.1986 | 0.2521 | -0.0055 | -0.2300 | 0.2190 | 0.0407 | -0.1545 | 0.2359 |
|  |  | **Moderate reliance** | -0.1044 | -0.1891 | -0.0198 | 0.1548 | 0.0602 | 0.2493 | -0.0757 | -0.1533 | 0.0019 | -0.0353 | -0.1166 | 0.0460 | 0.0050 | -0.0639 | 0.0739 |
|  |  | **Low reliance** | -0.1577 | -0.3509 | 0.0356 | 0.1287 | -0.1042 | 0.3616 | -0.0288 | -0.2233 | 0.1657 | 0.0191 | -0.1771 | 0.2153 | -0.0237 | -0.1863 | 0.1390 |
| Mid boreal | **Resident** | **High reliance** | 0.0182 | -0.0611 | 0.0974 | -0.0312 | -0.1049 | 0.0426 | 0.0139 | -0.0525 | 0.0803 | 0.0435 | -0.0434 | 0.1305 | -0.0133 | -0.0662 | 0.0396 |
|  |  | **Moderate reliance** | -0.0513 | -0.1891 | 0.0866 | -0.0274 | -0.1512 | 0.0963 | -0.0922 | -0.2040 | 0.0196 | 0.0670 | -0.0502 | 0.1842 | 0.0285 | -0.0716 | 0.1287 |
|  |  | **Low reliance** | 0.0448 | -0.1118 | 0.2015 | -0.0537 | -0.1946 | 0.0873 | 0.0201 | -0.1030 | 0.1432 | -0.0149 | -0.1790 | 0.1492 | -0.0495 | -0.1542 | 0.0552 |
|  |  | **Other invertebrates** | -0.0416 | -0.1598 | 0.0767 | 0.0389 | -0.0713 | 0.1491 | 0.0051 | -0.0941 | 0.1042 | 0.0152 | -0.0930 | 0.1234 | 0.0345 | -0.0618 | 0.1307 |
|  |  | **Plants** | 0.0549 | -0.1981 | 0.3080 | -0.1391 | -0.3655 | 0.0873 | 0.0251 | -0.1906 | 0.2408 | 0.0133 | -0.3352 | 0.3619 | -0.0419 | -0.2447 | 0.1608 |
|  | **Short-distance** | **Moderate reliance** | -0.0550 | -0.1138 | 0.0038 | 0.0214 | -0.0342 | 0.0770 | 0.0203 | -0.0283 | 0.0690 | -0.0175 | -0.0747 | 0.0398 | 0.0284 | -0.0183 | 0.0750 |
|  |  | **Low reliance** | -0.0215 | -0.1589 | 0.1159 | 0.0768 | -0.0476 | 0.2011 | -0.0375 | -0.1467 | 0.0716 | 0.0100 | -0.1349 | 0.1549 | 0.0405 | -0.0581 | 0.1391 |
|  |  | **Plants** | -0.0475 | -0.1352 | 0.0402 | 0.0377 | -0.0432 | 0.1185 | 0.0146 | -0.0572 | 0.0865 | -0.0584 | -0.1579 | 0.0410 | 0.0466 | -0.0389 | 0.1320 |
|  | **Long-distance** | **High reliance** | 0.0060 | -0.1512 | 0.1631 | 0.0157 | -0.1070 | 0.1385 | 0.0207 | -0.0848 | 0.1261 | 0.0396 | -0.0854 | 0.1645 | 0.0459 | -0.0523 | 0.1442 |
|  |  | **Moderate reliance** | 0.0373 | -0.0196 | 0.0941 | -0.0144 | -0.0684 | 0.0396 | -0.0501 | -0.0976 | -0.0027 | 0.0354 | -0.0189 | 0.0897 | -0.0027 | -0.0443 | 0.0389 |
|  |  | **Low reliance** | -0.0447 | -0.1493 | 0.0598 | 0.0027 | -0.0933 | 0.0986 | -0.0120 | -0.1005 | 0.0765 | 0.0461 | -0.0546 | 0.1468 | 0.0218 | -0.0631 | 0.1068 |
| South boreal | **Resident** | **High reliance** | 0.0095 | -0.0389 | 0.0578 | 0.0233 | -0.0244 | 0.0711 | 0.0066 | -0.0323 | 0.0455 | 0.0802 | 0.0098 | 0.1506 | 0.0142 | -0.0193 | 0.0478 |
|  |  | **Moderate reliance** | 0.0131 | -0.0618 | 0.0881 | 0.0127 | -0.0651 | 0.0906 | -0.0191 | -0.0811 | 0.0430 | 0.0330 | -0.0424 | 0.1083 | -0.0023 | -0.0580 | 0.0533 |
|  |  | **Low reliance** | 0.1181 | 0.0153 | 0.2210 | -0.0222 | -0.1201 | 0.0757 | 0.0744 | -0.0054 | 0.1542 | -0.0406 | -0.1975 | 0.1163 | -0.0421 | -0.1087 | 0.0246 |
|  |  | **Other invertebrates** | -0.0131 | -0.0816 | 0.0553 | 0.0183 | -0.0473 | 0.0840 | 0.0001 | -0.0527 | 0.0529 | -0.0040 | -0.0740 | 0.0659 | 0.0076 | -0.0426 | 0.0578 |
|  |  | **Plants** | 0.0079 | -0.1270 | 0.1428 | 0.0392 | -0.0967 | 0.1752 | -0.0547 | -0.1705 | 0.0610 | 0.0665 | -0.1622 | 0.2953 | 0.1018 | 0.0068 | 0.1969 |
|  | **Short-distance** | **Moderate reliance** | 0.0209 | -0.0183 | 0.0602 | 0.0040 | -0.0351 | 0.0431 | 0.0136 | -0.0191 | 0.0464 | 0.0011 | -0.0371 | 0.0392 | 0.0187 | -0.0131 | 0.0505 |
|  |  | **Low reliance** | -0.0055 | -0.0838 | 0.0727 | 0.0417 | -0.0356 | 0.1190 | -0.0282 | -0.0932 | 0.0368 | 0.0075 | -0.0780 | 0.0931 | 0.0240 | -0.0401 | 0.0882 |
|  |  | **Plants** | -0.0460 | -0.0991 | 0.0071 | 0.0593 | 0.0069 | 0.1117 | -0.0122 | -0.0555 | 0.0310 | -0.0583 | -0.1311 | 0.0145 | 0.0029 | -0.0353 | 0.0411 |
|  | **Long-distance** | **High reliance** | -0.0170 | -0.0937 | 0.0597 | -0.0097 | -0.0846 | 0.0653 | 0.0082 | -0.0515 | 0.0679 | 0.0179 | -0.0628 | 0.0986 | 0.0191 | -0.0377 | 0.0758 |
|  |  | **Moderate reliance** | -0.0092 | -0.0522 | 0.0339 | 0.0227 | -0.0192 | 0.0645 | -0.0050 | -0.0408 | 0.0308 | -0.0067 | -0.0566 | 0.0432 | 0.0039 | -0.0280 | 0.0358 |
|  |  | **Low reliance** | -0.0027 | -0.0645 | 0.0590 | 0.0340 | -0.0268 | 0.0948 | 0.0288 | -0.0198 | 0.0775 | 0.0059 | -0.0561 | 0.0679 | -0.0096 | -0.0571 | 0.0380 |

**Table S4. Covariate effects on bird functional groups estimated for average species body mass (second linear predictor).** Covariates are ln[moth biomass anomaly of previous year] of larval-overwintering moths, adult- / egg-overwintering moths, pupal-overwintering moths, and previous year anomaly of mean temperature and precipitation sum during bird breeding season in north-boreal, mid-boreal, and south-boreal regions in Finland. Lower and upper 99.8% confidence limits (LCI and UCI, respectively; adjusted for multiple testing calculated as 100 × (1 – (0.05/2×11))% = 99.8%) of the estimated covariate effects are given.

|  |  |  | Moth overwintering stage | | | | | | | | | Climatic covariates | | | | | |
| --- | --- | --- | --- | --- | --- | --- | --- | --- | --- | --- | --- | --- | --- | --- | --- | --- | --- |
| Zone  (Models) | **Migratory status** | **Caterpillar reliance/Diet** | **Larva** | | | **Adult/Egg** | | | **Pupa** | | | **Temperature** | | | **Precipitation** | | |
|  |  |  | **Estimate** | **LCI** | **UCI** | **Estimate** | **LCI** | **UCI** | **Estimate** | **LCI** | **UCI** | **Estimate** | **LCI** | **UCI** | **Estimate** | **LCI** | **UCI** |
| North boreal | **Resident** | **High reliance** | -0.0106 | -0.1133 | 0.0921 | -0.0684 | -0.1933 | 0.0565 | 0.1199 | 0.0288 | 0.2110 | 0.0432 | -0.0487 | 0.1351 | -0.0232 | -0.0970 | 0.0506 |
|  |  | **Moderate reliance** | -0.0670 | -0.1793 | 0.0453 | 0.0771 | -0.0490 | 0.2032 | -0.0490 | -0.1421 | 0.0441 | -0.0224 | -0.1250 | 0.0801 | -0.0358 | -0.1205 | 0.0489 |
|  |  | **Low reliance** | 0.0286 | -0.0255 | 0.0826 | 0.0118 | -0.0484 | 0.0719 | -0.0357 | -0.0833 | 0.0118 | 0.0572 | 0.0066 | 0.1077 | 0.0064 | -0.0343 | 0.0471 |
|  |  | **Other invertebrates** | -0.0432 | -0.1326 | 0.0462 | 0.0414 | -0.0794 | 0.1622 | -0.0037 | -0.0864 | 0.0790 | -0.0458 | -0.1284 | 0.0369 | 0.0566 | -0.0111 | 0.1243 |
|  |  | **Plants** | 0.0264 | -0.0665 | 0.1194 | 0.0233 | -0.0675 | 0.1142 | -0.0417 | -0.1138 | 0.0304 | 0.0159 | -0.0675 | 0.0994 | -0.0235 | -0.0860 | 0.0391 |
|  | **Short-distance** | **Moderate reliance** | -0.0412 | -0.0974 | 0.0150 | 0.0274 | -0.0354 | 0.0903 | -0.0120 | -0.0603 | 0.0363 | -0.0341 | -0.0821 | 0.0139 | -0.0252 | -0.0659 | 0.0156 |
|  |  | **Low reliance** | 0.0173 | -0.0468 | 0.0813 | 0.0089 | -0.0625 | 0.0803 | -0.0172 | -0.0715 | 0.0371 | -0.0014 | -0.0587 | 0.0559 | -0.0445 | -0.0917 | 0.0028 |
|  |  | **Plants** | 0.0009 | -0.1067 | 0.1085 | 0.0219 | -0.1001 | 0.1439 | 0.0088 | -0.0872 | 0.1048 | -0.0342 | -0.1334 | 0.0651 | -0.0253 | -0.1057 | 0.0550 |
|  | **Long-distance** | **High reliance** | 0.0196 | -0.0280 | 0.0673 | -0.0037 | -0.0600 | 0.0525 | 0.0177 | -0.0249 | 0.0603 | 0.0027 | -0.0373 | 0.0427 | -0.0069 | -0.0418 | 0.0279 |
|  |  | **Moderate reliance** | 0.0269 | -0.0458 | 0.0995 | -0.0047 | -0.0859 | 0.0764 | 0.0178 | -0.0434 | 0.0790 | 0.0086 | -0.0530 | 0.0702 | -0.0122 | -0.0637 | 0.0393 |
|  |  | **Low reliance** | -0.0124 | -0.0712 | 0.0463 | 0.0268 | -0.0415 | 0.0951 | 0.0155 | -0.0338 | 0.0649 | -0.0110 | -0.0614 | 0.0394 | -0.0126 | -0.0521 | 0.0270 |
| Mid boreal | **Resident** | **High reliance** | -0.0164 | -0.0639 | 0.0310 | -0.0607 | -0.1058 | -0.0156 | 0.0587 | 0.0183 | 0.0991 | -0.0395 | -0.0818 | 0.0029 | 0.0107 | -0.0303 | 0.0516 |
|  |  | **Moderate reliance** | -0.0462 | -0.1636 | 0.0711 | 0.0423 | -0.0763 | 0.1609 | 0.0148 | -0.0882 | 0.1177 | -0.0259 | -0.1251 | 0.0733 | 0.0469 | -0.0511 | 0.1450 |
|  |  | **Low reliance** | 0.0044 | -0.0245 | 0.0333 | 0.0020 | -0.0252 | 0.0291 | 0.0063 | -0.0163 | 0.0289 | -0.0054 | -0.0306 | 0.0198 | 0.0038 | -0.0195 | 0.0270 |
|  |  | **Other invertebrates** | 0.0021 | -0.0367 | 0.0409 | -0.0066 | -0.0436 | 0.0303 | 0.0092 | -0.0251 | 0.0434 | -0.0004 | -0.0354 | 0.0346 | -0.0308 | -0.0647 | 0.0030 |
|  |  | **Plants** | 0.0004 | -0.0889 | 0.0897 | -0.0374 | -0.1081 | 0.0332 | -0.0189 | -0.0898 | 0.0520 | -0.0211 | -0.0966 | 0.0544 | -0.0654 | -0.1340 | 0.0032 |
|  | **Short-distance** | **Moderate reliance** | 0.0349 | -0.0055 | 0.0753 | -0.0369 | -0.0749 | 0.0011 | 0.0240 | -0.0086 | 0.0566 | 0.0093 | -0.0244 | 0.0430 | -0.0209 | -0.0530 | 0.0111 |
|  |  | **Low reliance** | 0.0086 | -0.0394 | 0.0567 | -0.0373 | -0.0835 | 0.0089 | -0.0134 | -0.0520 | 0.0251 | 0.0151 | -0.0288 | 0.0589 | 0.0109 | -0.0291 | 0.0509 |
|  |  | **Plants** | -0.0072 | -0.1024 | 0.0879 | -0.0703 | -0.1594 | 0.0188 | 0.0219 | -0.0559 | 0.0998 | 0.0736 | -0.0164 | 0.1636 | 0.0466 | -0.0389 | 0.1320 |
|  | **Long-distance** | **High reliance** | 0.0195 | -0.0101 | 0.0491 | -0.0133 | -0.0419 | 0.0153 | -0.0100 | -0.0352 | 0.0152 | 0.0046 | -0.0209 | 0.0301 | -0.0027 | -0.0280 | 0.0226 |
|  |  | **Moderate reliance** | -0.0092 | -0.0570 | 0.0386 | -0.0080 | -0.0538 | 0.0379 | 0.0292 | -0.0105 | 0.0689 | 0.0187 | -0.0234 | 0.0608 | -0.0056 | -0.0440 | 0.0327 |
|  |  | **Low reliance** | -0.0046 | -0.0327 | 0.0235 | 0.0006 | -0.0259 | 0.0272 | -0.0070 | -0.0310 | 0.0171 | -0.0026 | -0.0266 | 0.0214 | -0.0102 | -0.0334 | 0.0130 |
| South boreal | **Resident** | **High reliance** | -0.0199 | -0.0497 | 0.0100 | -0.0013 | -0.0305 | 0.0279 | 0.0121 | -0.0130 | 0.0371 | -0.0228 | -0.0508 | 0.0052 | 0.0106 | -0.0155 | 0.0366 |
|  |  | **Moderate reliance** | 0.0005 | -0.0581 | 0.0591 | 0.0053 | -0.0540 | 0.0646 | -0.0051 | -0.0552 | 0.0449 | -0.0283 | -0.0790 | 0.0224 | -0.0091 | -0.0613 | 0.0430 |
|  |  | **Low reliance** | 0.0123 | -0.0150 | 0.0395 | 0.0106 | -0.0160 | 0.0371 | -0.0045 | -0.0271 | 0.0181 | 0.0133 | -0.0114 | 0.0379 | 0.0117 | -0.0116 | 0.0350 |
|  |  | **Other invertebrates** | -0.0002 | -0.0224 | 0.0220 | 0.0036 | -0.0183 | 0.0255 | 0.0039 | -0.0144 | 0.0222 | 0.0013 | -0.0180 | 0.0205 | 0.0009 | -0.0185 | 0.0203 |
|  |  | **Plants** | -0.0314 | -0.0909 | 0.0282 | 0.0136 | -0.0437 | 0.0708 | -0.0164 | -0.0670 | 0.0342 | 0.0345 | -0.0247 | 0.0936 | 0.0049 | -0.0475 | 0.0573 |
|  | **Short-distance** | **Moderate reliance** | -0.0375 | -0.0684 | -0.0066 | 0.0155 | -0.0151 | 0.0460 | -0.0049 | -0.0310 | 0.0212 | 0.0012 | -0.0255 | 0.0279 | -0.0017 | -0.0277 | 0.0243 |
|  |  | **Low reliance** | -0.0007 | -0.0182 | 0.0167 | -0.0078 | -0.0256 | 0.0099 | -0.0021 | -0.0174 | 0.0132 | -0.0027 | -0.0185 | 0.0131 | -0.0026 | -0.0182 | 0.0129 |
|  |  | **Plants** | 0.0761 | 0.0350 | 0.1171 | -0.0900 | -0.1306 | -0.0493 | 0.0007 | -0.0337 | 0.0350 | 0.0862 | 0.0469 | 0.1256 | -0.0185 | -0.0564 | 0.0194 |
|  | **Long-distance** | **High reliance** | -0.0224 | -0.0462 | 0.0013 | 0.0158 | -0.0074 | 0.0391 | 0.0042 | -0.0151 | 0.0234 | -0.0088 | -0.0293 | 0.0117 | -0.0049 | -0.0246 | 0.0148 |
|  |  | **Moderate reliance** | 0.0010 | -0.0355 | 0.0374 | -0.0035 | -0.0392 | 0.0322 | 0.0061 | -0.0244 | 0.0366 | 0.0407 | 0.0058 | 0.0755 | 0.0184 | -0.0136 | 0.0505 |
|  |  | **Low reliance** | -0.0070 | -0.0283 | 0.0143 | 0.0024 | -0.0187 | 0.0235 | 0.0016 | -0.0161 | 0.0193 | -0.0133 | -0.0323 | 0.0057 | -0.0212 | -0.0397 | -0.0028 |


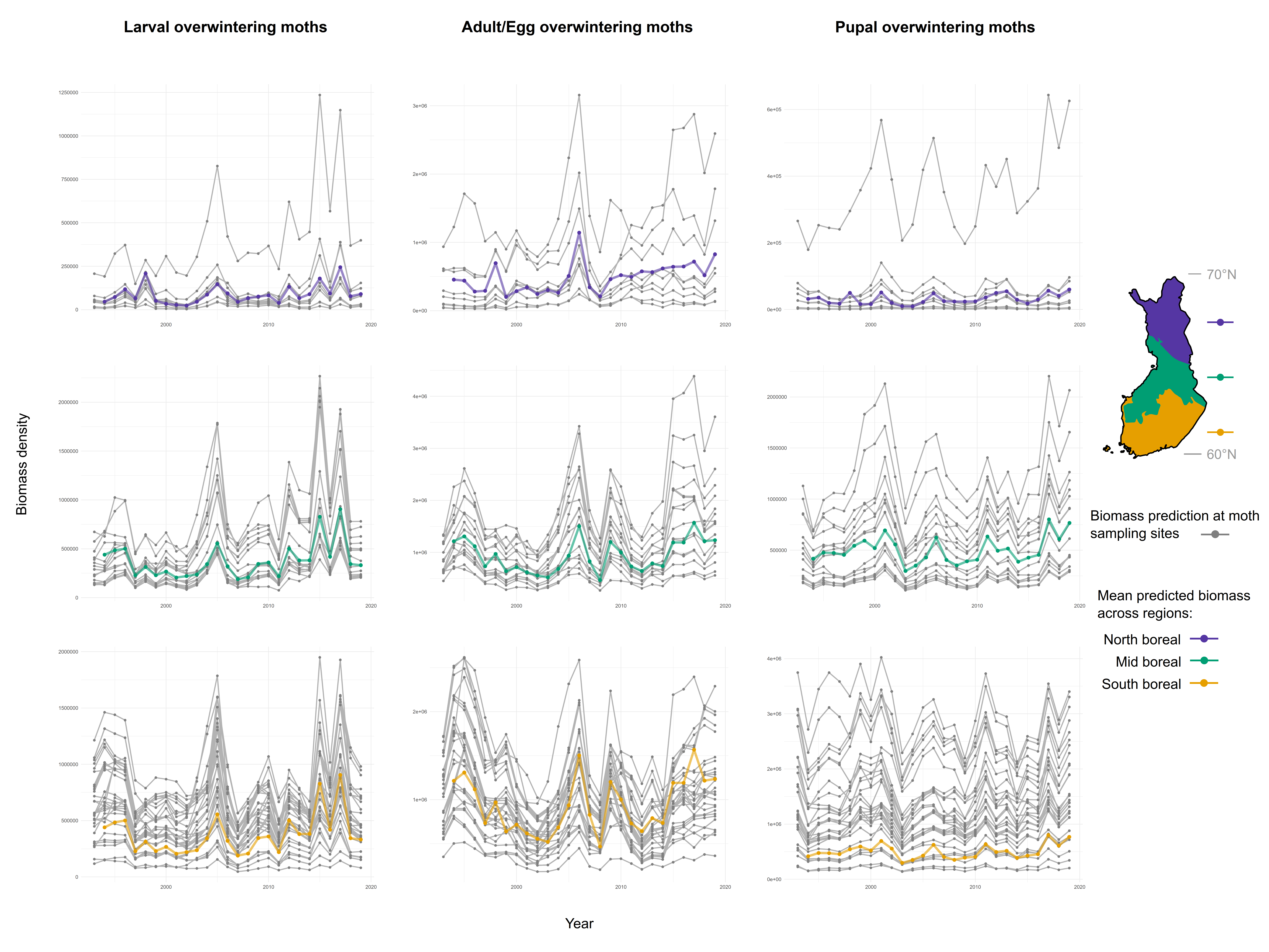


**Fig. S1. Time series of the predicted biomass of moths overwintering** as (a) larva, (b) adult or egg, or (c) pupa in the north-boreal (violet), mid-boreal (green) and south-boreal (yellow) regions in Finland, separately for all moth sampling sites. The thick lines indicate the region-specific means of predicted biomass.


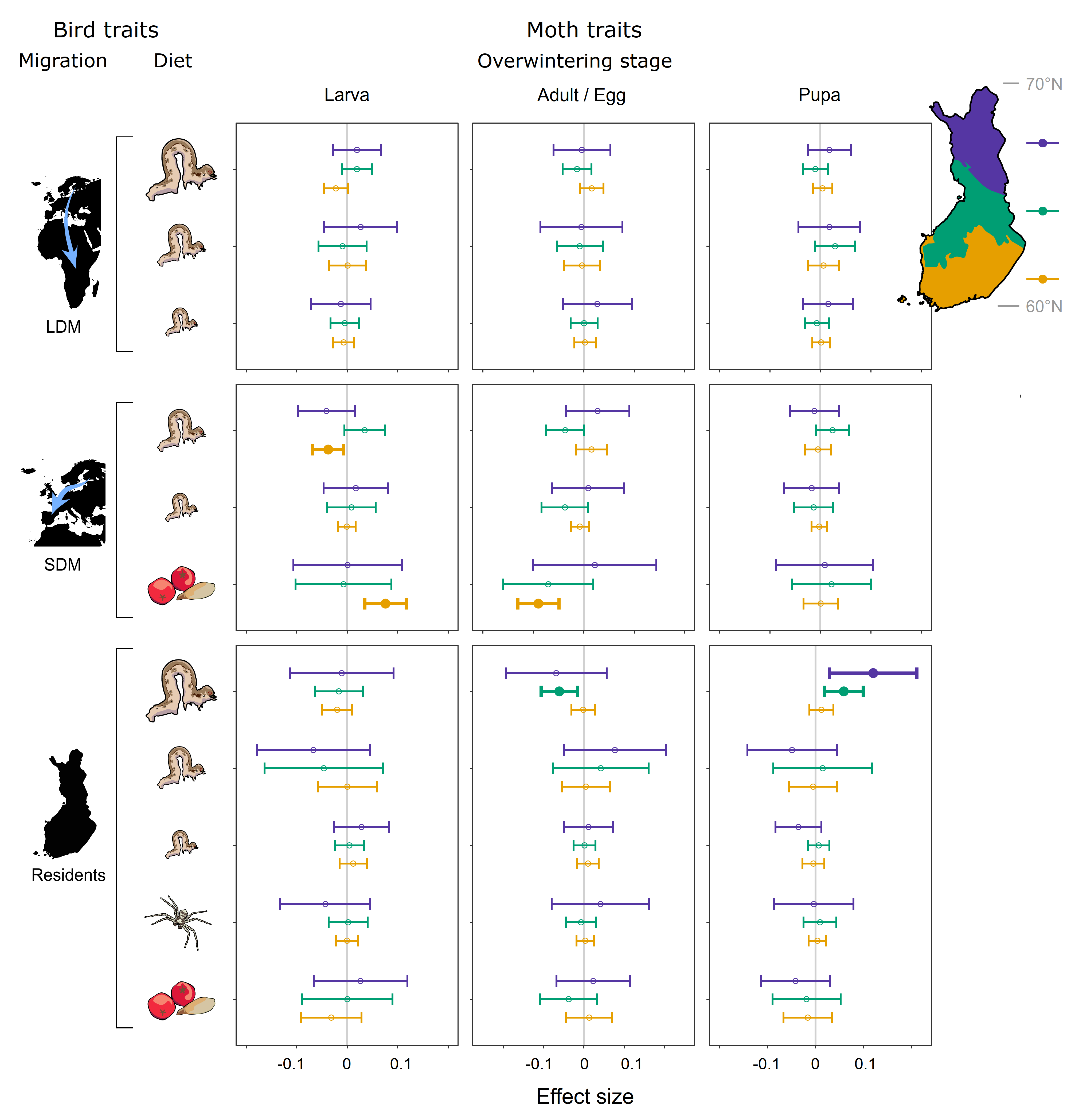


**Fig. S2.** **Estimated association of ln-transformed moth biomass anomaly of the previous year** of larval-overwintering moths (left column), adult- / egg-overwintering moths (middle column), pupal-overwintering moths (right column) **with** **average species body mass of bird functional groups** (rows) in north-boreal (violet), mid-boreal (green), and south-boreal (orange) regions in Finland. Bird migratory status consists of Residents, short-distance migrants (SDM), and long-distance migrants (LDM). For bird diet, the reliance of birds on caterpillars is indicated by caterpillar size (i.e., larger caterpillar links to higher reliance), the spider represents reliance on other invertebrates, and the rowanberries and the pine seed represent reliance on plants as food. Points stand for the estimated effect size, while whiskers are confidence intervals adjusted for multiple testing calculated as 100 × (1 – (0.05/2×11))% = 99.8%. Significant effects (α = 0.002) are shown with bold whiskers and filled points.


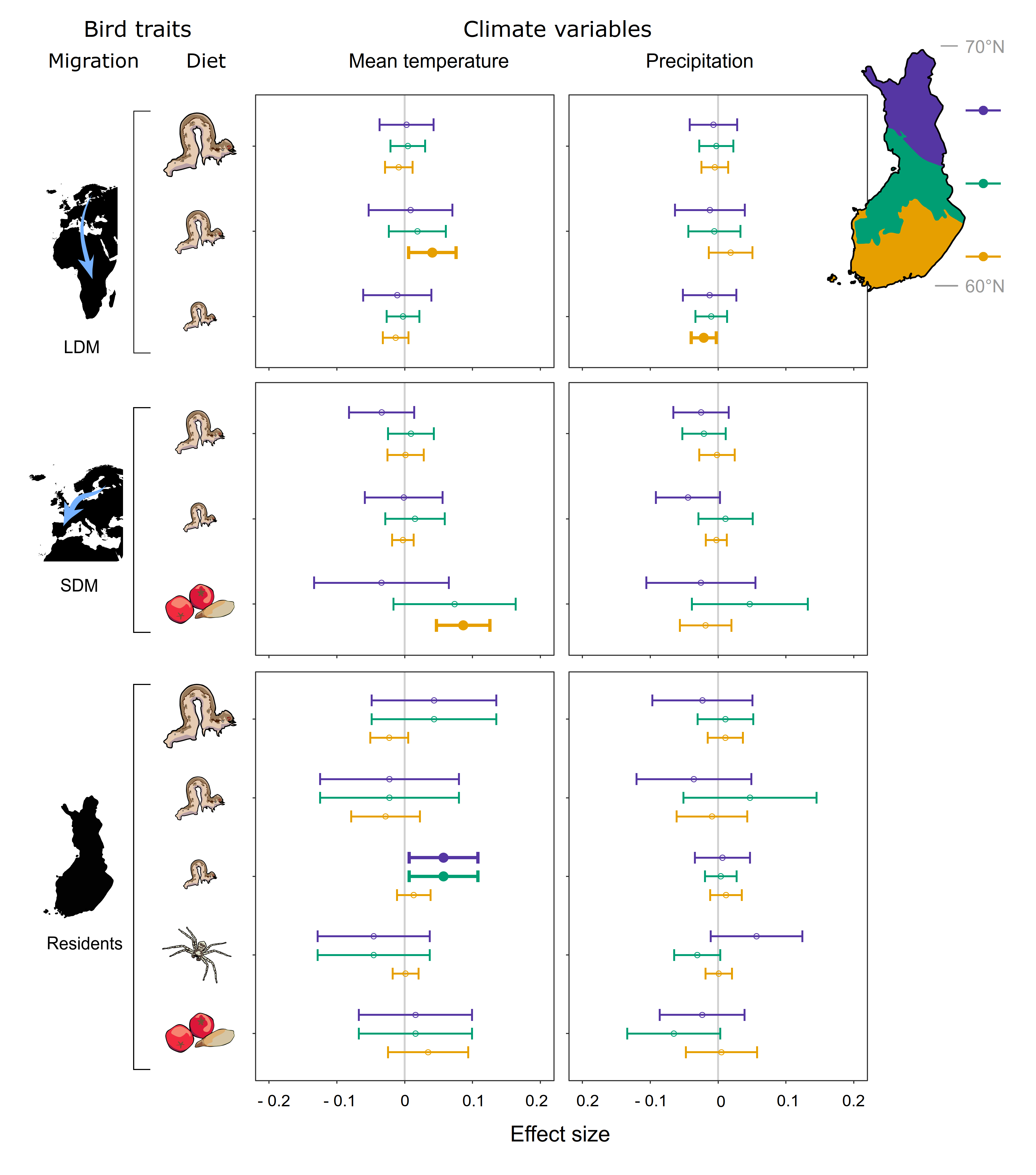


**Fig. S3. Estimated association of previous year anomaly of mean temperature** (left column) **and precipitation sum** (right column) **during bird breeding season with average species body mass of bird functional groups** (rows) in north-boreal (violet), mid-boreal (green), and south-boreal (orange) regions in Finland. Bird migratory status consists of Residents, short-distance migrants (SDM), and long-distance migrants (LDM). See Fig. S2 for information on symbols, points and whiskers.


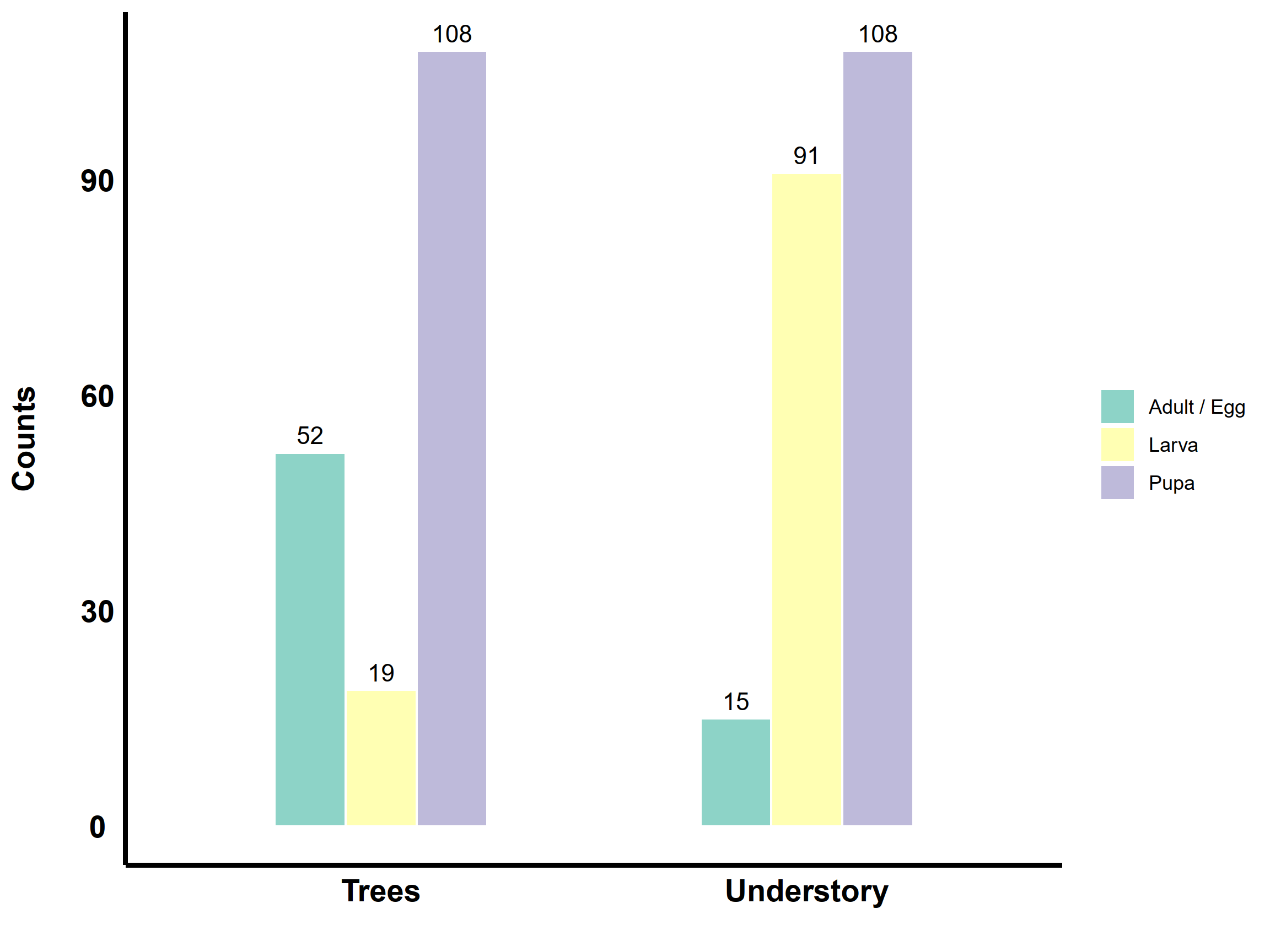


**Fig. S4.** Host plant growth form for moths overwintering in different life stages. Only moth species considered in this study are included. Numbers above the bars indicate species numbers.
