# Supplementary material for: Evidence for bottom‐up effects of moth abundance on forest birds in the north‐boreal zone alone
Source: Ecol Lett. 2024 Dec 31;27(12):e14467. doi: 10.1111/ele.14467 (PMC11686949; doi:10.1111/ele.14467)
Supplement: Supplementary file 4 — Appendix S1. [file ELE-27-0-s005.docx]

**Appendix S1**

**Moth models and interpolation**

We modelled spatio-temporal biomass variation in the three moth functional groups in 1993-2018 by using a similar VAST model as explained in the main text for birds, except that the model included only a single linear predictor because the moth functional groups had an essentially 100% occurrence across years and sites. The annual total biomasses of moth functional groups for each year and trap location were the response variables. The VAST analysis generated specific biomass predictions for moth functional groups across 85 spatial knot locations, corresponding to the number of trap sites. To extend these estimates across Finland, we interpolated the values from each knot. Using the 5 nearest neighbour method, we created a Voronoi diagram of these knots, subsequently rasterizing the Voronoi mesh at a 10 km resolution. From these 10 km rasters (one raster for each year), we extracted annual moth biomass estimates at the precise locations of the bird observation sites. We also checked the associated uncertainty of biomass interpolation, the interpolated biomass values fluctuating highly synchronously both within and across regions (see Fig. S1). Then, the annual biomasses were ln-transformed, and the mean of the site was deduced to create yearly ln-anomalies of moth biomass for each bird observation site. The ln-transformed biomass anomalies of the three moth functional groups from the previous year were set as covariates in the bird analyses.
