# Supplementary material for: Evidence for bottom‐up effects of moth abundance on forest birds in the north‐boreal zone alone
Source: Ecol Lett. 2024 Dec 31;27(12):e14467. doi: 10.1111/ele.14467 (PMC11686949; doi:10.1111/ele.14467)
Supplement: Supplementary file 5 — Appendix S2. [file ELE-27-0-s004.docx]

**Appendix S2**

**Sensitivity analyses for appropriate time lag between moths and birds**

We ran three sets of analyses for the north-boreal region for the period 1995-2019, each using different time lags. T-0 model utilized no time lag, relying on the current year moth biomass, the T-1 model employed a one-year time lag, utilizing moth biomass from the previous year, while the T-2 model incorporated a two-year time lag, utilizing moth biomass from two years ago. Model settings and the climatic covariates remained the same in all models. After comparing the outputs of these three models, we found that model T-1, which includes a one-year time lag, performed the best in terms of AIC (ΔAIC=8.6 between the best two time lags T-1 and T0, in favour of T-1; Table A). While the percentage of deviance explained by the models remained relatively consistent across all three models, model T-1 reached the lowest AIC value. To further evaluate the appropriateness of our T-1 time lag assumption, we compared and plotted the effect sizes for the associations between moth covariates and 11 functional groups (Fig. A). The fixed effects of the three time lag models are mostly consistent, with similar patterns observed across functional groups. However, it is worth noting that there are a few instances where the effects differ among the time lags, indicating potential variations in the relationship between moth biomass and bird populations over time.

**Table A. Model performance assessment using tools provided in the VAST R package.** The proportion of explained deviance is calculated as one minus the deviance of the target model divided by the deviance of the null model. Whilst there are no meaningful differences in proportions of explained deviance among the three models, the ΔAIC=8.6 with the second-best model T-0.

| *Zone* | *Models* | *Deviance of target model* | *Deviance of null model* | *Proportion of explained deviance* | *ΔAICs** |
| --- | --- | --- | --- | --- | --- |
|  | T0 | 92400.04 | 120295.7 | 0.2318 | 8.6 |
| North boreal | T-1 | 92415.27 | 120295.7 | 0.2317 | 0.0 |
|  | T-2 | 92395.7 | 120295.7 | 0.2319 | 47.7 |

*The ΔAICs are calculated as the difference between AIC values of each model and the AIC value of the best model (T-0).


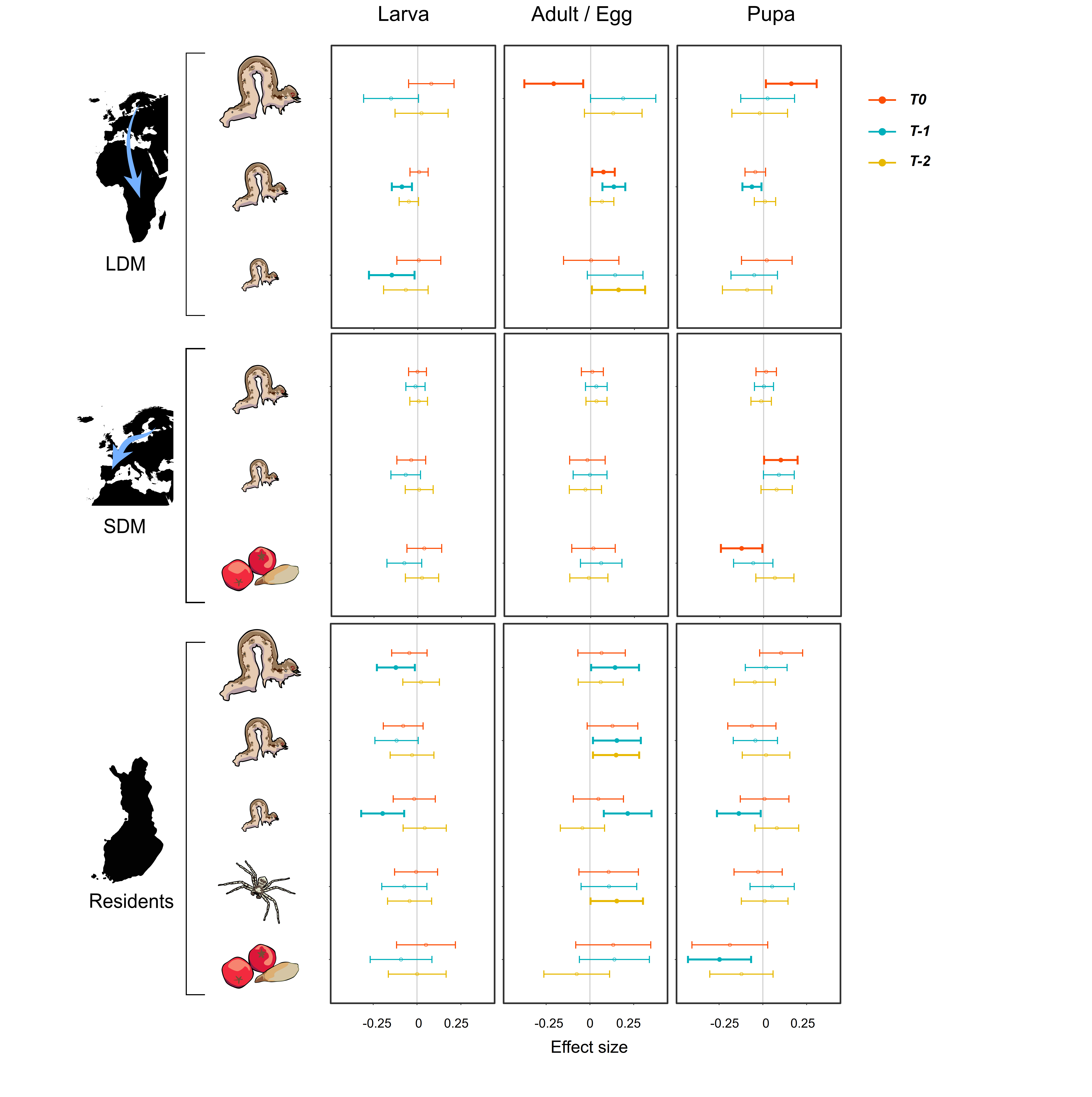


**Fig. A.** Covariate effect sizes for three different models including different time-lags for moths: T0, current year moth biomass; T-1, previous year moth biomass; T-2, moth biomass from two years ago.
