# Supplementary material for: Evidence for bottom‐up effects of moth abundance on forest birds in the north‐boreal zone alone
Source: Ecol Lett. 2024 Dec 31;27(12):e14467. doi: 10.1111/ele.14467 (PMC11686949; doi:10.1111/ele.14467)
Supplement: Supplementary file 6 — Appendix S3. [file ELE-27-0-s007.docx]

**Appendix S3**

**VAST model parameters and settings**

We configured the VAST model as a Poisson-link delta model, which closely approximates a Tweedie distribution to enhance computational efficiency and to facilitate easy interpretation of covariate effects (Thorson 2018). In delta models, the probability distribution for the biomass data *b(s, c, t)* is broken down into occurrence probability of functional group *c*, at location *s* in year *t* (i.e., at least one species belonging to group *c* is present at site *s* in year *t*), *r₁(s, c, t)*, and biomass of functional group *c*, at location *s,* in year *t* conditional on occurrence, *r₂(s, c, t)*. We specified Gamma distribution for bird biomass, *b*, as

$\Pr\left( b_{s,c,t}=B \right)=\left\{ \begin{aligned} 1-r_{1}(s, c, t) if B=0 \\ r_{1}(s, c, t)\times gamma \left\{ B|r_{2}(s, c, t) ,\sigma_{m}^{2} \left( c \right) \right\} if B> 0 \end{aligned} \right.$(S3.1)

where $\sigma_{m}^{2}$ is the residual variance in biomass.

With the Poisson-link configuration, we can express *r₁(s, c, t)* as a function of density, *N(s, c, t)* (i.e., the number of individuals per unit area), and *r₂(s, c, t)* as a function of both *N(s, c, t)* and average species body mass, *W(s, c, t)* (i.e., the mean body mass of a species in group *c*), as follows (Thorson 2018, 2019):

$r_{1}\left( s,c,t \right)=1-\exp\left( -a_{i} \times\exp\left( N\left( s,c,t \right) \right) \right)$ (S3.2)

$r_{2}\left( s,c,t \right) = \frac{a_{i} \times\exp\left( N\left( s,c,t \right) \right)}{r_{1} \left( s,c,t \right)} \times\exp\left( W\left( s,c,t \right) \right)$ (S3.3)

In equations S3.2 and S3.3, $a_{i}$ represents the sampling area (km^2^) used to estimate the densities of functional groups. We assumed that birds were observed within a 75 m radius from the observation points, so *a_i_* was calculated as [0.15 km] × [the length of the habitat strip in question (km)] for line transects, and π × [0.075 km]^2^ for point counts). Although we determined the observation radius subjectively, it does not affect our conclusions as the radius only affects the intercept of density estimates (e.g., biomass per unit area), rather than the relative differences among sampling sites and years, with the relative differences being important in this work. In the Poisson-link configuration, density *N(s, c, t)* and the average species body mass *W(s, c, t)* are modelled through two linear predictors, both of which include terms for spatial and spatio-temporal variation. The linear predictor for *N(s, c, t)* is

$$\ln(N\left( s,c,t \right))= \beta_{1} \left( c, t \right) + \underset{Spatial variation}{\underbrace{\sum_{f=1}^{n_{\omega1}} \boldsymbol{L}_{\omega1}\left( c,f \right)\omega_{1}\left( s, f \right)}} + \underset{Spatio-temporal variation}{\underbrace{\sum_{f=1}^{n_{\varepsilon1}} \boldsymbol{L}_{\varepsilon1}\left( c,f \right)\varepsilon_{1}\left( s,t, f \right)}} + \underset{Covariates}{\underbrace{\sum_{j=1}^{5} \gamma_{j}x_{j}(s,t)}} + \underset{Overdispersion}{\underbrace{\sum_{f=1}^{n_{\eta1}} \boldsymbol{L}_{\eta1}\left( c,f \right)\eta_{1}\left( \upsilon,f \right)}} + \underset{Bird census type}{\underbrace{\sum_{k=1}^{2} \boldsymbol{\lambda}_{1}\left( k \right)Q\left( i,k \right)}}$$

(S3.4)

and the linear predictor for *W(s, c, t)* is

$$\ln\left( W\left( s,c,t \right) \right)= \beta_{2} \left( c,t \right) + \underset{Spatial variation}{\underbrace{\sum_{f=1}^{n_{\omega2}} \boldsymbol{L}_{\omega2}\left( c,f \right)\omega_{2}\left( s, f \right)}} + \underset{Spatio-temporal variation}{\underbrace{\sum_{f=1}^{n_{\varepsilon2}} \boldsymbol{L}_{\varepsilon2}\left( c,f \right)\varepsilon_{2}\left( s,t, f \right)}} + \underset{Covariates}{\underbrace{\sum_{j=1}^{5} \delta_{j}x_{j}\left( s, t \right)}} + \underset{Overdispersion}{\underbrace{\sum_{f=1}^{n_{\eta2}} \boldsymbol{L}_{\eta2}\left( c,f \right)\eta_{2}\left( \upsilon,f \right)}} + \underset{Bird census type}{\underbrace{\sum_{k=1}^{2} \boldsymbol{\lambda}_{2}\left( k \right)Q\left( i,k \right)}}$$

(S3.5)

In equations S3.4 and S3.5, $\beta_{p} \left( c,t \right)$ (*p*={1, 2}) is the intercept for either the density (*p*=1; fixed effect for each year with a first-order temporal autoregressive process [AR1]) or the average species body mass (*p*=2; independent fixed effect for each year with no temporal structure) for functional group *c* in year *t*. $\omega_{p}\left( s,f \right)$ and $\varepsilon_{p}\left( s,t, f \right)$ are vectors of spatial and spatio-temporal random effects, respectively, for factor *f*, at site *s* and year *t*. Parameter $\gamma_{j}$ is the estimated effect of covariate *j* (*j* = {ln[previous year biomass anomaly of moth group *q*], anomaly in mean temperature, anomaly in total precipitation}; *q*={adult/egg overwintering, larval overwintering, pupal overwintering}) on density, *x_j_*(*s,t*) being the value of covariate *j* at site *s* in year *t*. Similarly, *δ_j_* is the effect of covariate *j* on average species body mass. $\eta_{p}\left( \upsilon,f \right)$ represents random variation in overdispersion for each factor (totally ${n_{\eta}}_{p}$ factors modelling overdispersion at the level of route identities). $\boldsymbol{\lambda}_{p}\left( k \right)$ represents the impact of bird census type covariates *Q* for each sample *i* and sampling method *k* (*k*={line transect, point count}) to take into account potential bird detection differences between the sampling methods. $\boldsymbol{L}_{\omega p}\left( c,f \right)$, $\boldsymbol{L}_{\varepsilon p}\left( c,f \right)$ and $\boldsymbol{L}_{\eta p}\left( c,f \right)$ are loadings matrices for spatial, spatio-temporal and overdispersion covariation, respectively, that define the responses of bird functional groups to the $n_{\omega p}$, $n_{\varepsilon p}$ and $n_{\eta p}$ latent factors. We set the number of latent factors for spatial $\boldsymbol{L}_{\omega p}\left( c,f \right)$, spatio-temporal $\boldsymbol{L}_{\varepsilon p}\left( c,f \right)$, and overdispersion variation $\boldsymbol{L}_{\eta p}\left( c,f \right)$ in both linear predictors to the maximum that allowed for model convergence. Consequently, the models included five, four, and two latent factors, in north-boreal, mid-boreal, and south-boreal regions, respectively.

We adopted the "grid" method for spatial modelling, utilizing 1500 spatial knots, with the exception of 800 in the south-boreal model to facilitate convergence. The spatial knots were positioned at the centroids of grid cells containing at least one sampling site, creating a systematically distributed network of knots, one knot being assigned to each sample. Spatial ($\omega_{p}\left( s,f \right)$) and spatio-temporal ($\varepsilon_{p}\left( s,t, f \right)$) random effects were modelled at the knot locations by Gaussian random fields, following multivariate normal (MVN) probability distributions. Stochastic partial differential equations (SPDE; Lindgren et al., 2011) were used to approximate these Gaussian random fields, by using the R-INLA package (Lindgren & Rue 2015; Thorson et al. 2015).

We defined spatial variation as constant over time (Eq.S3.6), and spatio-temporal variation without temporal structure (i.e., independent among years; Eq.S3.7)

$\omega_{p}\left( s,f \right)\sim MVN\left( 0,\boldsymbol{R}_{\boldsymbol{p}} \right)$ (S3.6)

$\varepsilon_{p}\left( s,f,t \right)\sim\left\{ \begin{aligned} MVN\left( 0,\boldsymbol{R}_{\boldsymbol{p}} \right) \text{if }\text{t = }t_{1} \\ MVN\left( \varepsilon_{p}\left( s,f,t-1 \right),\boldsymbol{R}_{p} \right)\text{ if }\text{t > }t_{1} \end{aligned} \right.$ (S3.7)

where ***R****_p_* is a spatial correlation matrix approximating spatial covariation in linear predictor *p*, among sites *s* (*s*= 1, …, *n_sites_*; *n_sites_* equals 3571 in north-boreal, 4885 in mid-boreal, 8199 in south-boreal). Specifying a variance of 1 for spatial and spatio-temporal Gaussian random fields ($\sigma_{\omega}^{2}$ and $\sigma_{\varepsilon}^{2}$, respectively) ensures that the covariance among categories is defined by the loadings matrix for that term. To model the decay of spatial correlation with increasing distance, we used a Matèrn correlation function with a smoothness parameter set to *v* = 1. We also estimated a rate parameter *κ_p_* to define the distance at which locations are no longer correlated, and an estimated matrix **H** to account for the asymmetry in decorrelation distance across different directions (Thorson et al., 2016; Thorson & Barnett, 2017).

We set functional-group-specific intercepts $\beta_{p}\left( c, t \right)$, variance in spatial ($\sigma_{\omega}^{2}$) and spatio-temporal ($\sigma_{\varepsilon}^{2}$) variation, factor loadings matrices ($\boldsymbol{L}_{\omega p}$, $\boldsymbol{L}_{\varepsilon p}$, $\boldsymbol{L}_{\eta p}$), effect of environmental covariates $\gamma_{j}$ and *δ_j_*, bird census type covariates $\boldsymbol{\lambda}_{p}\left( k \right)$, the parameters governing the geometric anisotropy (**H**) and decorrelation distance (*к*) in the Matèrn correlation function as fixed effects. Spatial $\omega_{p}\left( s,f \right)$, spatio-temporal $\varepsilon_{p}\left( s,f,t \right)$, and overdispersion (i.e., census route identity) $\eta_{p}\left( \upsilon,f \right)$ factors were treated as random effects.

**References**

Lindgren, F. & Rue, H. (2015). Bayesian Spatial Modelling with R-INLA. *J Stat Softw*, 63, 1–25.

Lindgren, F., Rue, H. & Lindström, J. (2011). An explicit link between Gaussian fields and Gaussian Markov random fields: the stochastic partial differential equation approach. *J R Stat Soc Series B Stat Methodol*, 73, 423–498.

Thorson, J. T. (2019). Guidance for decisions using the Vector Autoregressive Spatio-Temporal (VAST) package in stock, ecosystem, habitat and climate assessments. *Fish. Res.*, 210, 143-161.

Thorson, J. T. (2018) Three problems with the conventional delta-model for biomass sampling data, and a computationally efficient alternative. *Can. J. Fish. Aquat. Sci.*, 75(9), 1369–1382.

Thorson, J.T. & Barnett, L.A.K. (2017). Comparing estimates of abundance trends and distribution shifts using single- and multispecies models of fishes and biogenic habitat. *ICES J. Mar. Sci.*, 74, 1311–1321.

Thorson, J.T., Ianelli, J.N., Larsen, E.A., Ries, L., Scheuerell, M.D., Szuwalski, C., *et al.* (2016). Joint dynamic species distribution models: a tool for community ordination and spatio-temporal monitoring. *Glob. Ecol. Biogeogr.*, 25, 1144–1158.

Thorson, J.T., Skaug, H.J., Kristensen, K., Shelton, A.O., Ward, E.J., Harms, J.H., *et al.* (2015). The importance of spatial models for estimating the strength of density dependence. *Ecology*, 96, 1202–1212.
