# Supplementary material for: Evidence for bottom‐up effects of moth abundance on forest birds in the north‐boreal zone alone
Source: Ecol Lett. 2024 Dec 31;27(12):e14467. doi: 10.1111/ele.14467 (PMC11686949; doi:10.1111/ele.14467)
Supplement: Supplementary file 7 — Appendix S4. [file ELE-27-0-s006.docx]

**Appendix S4**

**Correction for multiple testing and probability calculations**

We applied corrections to the alpha level to mitigate the risk of Type I errors (false positives) arising from conducting multiple statistical tests. We have three sets of independent models (i.e., a separate model for each of the north-boreal, mid-boreal, and south-boreal regions) and 11 tests within each moth functional group within each model, this set of 11 tests being our unit of assessment of bottom-up effects. Hence, we corrected the alpha level to 11 tests, so that the corrected confidence level for confidence interval (CI) calculation becomes 100 × 0.998 = 99.8%. Consequently, we use the quantile of 2.88 instead of 1.96 as the multiplier of the standard error of an effect size when calculating CIs for the estimated effects. We considered parameters with 99.8% CIs excluding zero to be statistically significant.

To assess the probability of finding the observed number of high effect sizes by chance, we calculated the probability of finding effects with sizes equal to or greater that the lowest effect size that was statistically significant in the strict sense (i.e., the CI did not encompass zero) within each moth-group-region combination. We did this by establishing an empirical probability distribution of effect sizes using the absolute values of all the effects from all our models. Then, we applied the empirical cumulative distribution function ‘ecdf’ in R to that distribution and used the empirical cumulative distribution function for quantifying the probability of observing a certain number of effect sizes equal or stronger in absolute value than the threshold specific for the combination of region and moth functional group in question, with the further condition that the CIs of effects included in the consideration only marginally encompassed zero. We calculated the probability as follows:

$$\boldsymbol{P}\mathbf{=}{\mathbf{(1-}\boldsymbol{F}\mathbf{(}\left[ \boldsymbol{moth-group}\mathbf{-}\boldsymbol{region specific} \boldsymbol{threshold} \right]\mathbf{))}}^{\boldsymbol{n}_{\boldsymbol{high} \boldsymbol{effect} \boldsymbol{sizes}}}$$

where F is the empirical cumulative distribution function and n_high effect sizes_ is the number of effect sizes as high or higher than the threshold value. We had five instances for which we could calculate the probabilities, because at least one significant effect per moth-group per region was required for this approach (cf. Fig. 3).
